# ProfhEX: AI-based platform for small molecules liability profiling

**DOI:** 10.1186/s13321-023-00728-6

**Published:** 2023-06-09

**Authors:** Filippo Lunghini, Anna Fava, Vincenzo Pisapia, Francesco Sacco, Daniela Iaconis, Andrea Rosario Beccari

**Affiliations:** 1 EXSCALATE, Dompé Farmaceutici SpA, Via Tommaso de Amicis 95, 80123 Naples, Italy; 2 Professional Service Department, SAS Institute, Via Darwin 20/22, 20143 Milan, Italy

**Keywords:** Virtual screening, Liability profiling, Polypharmacology, Machine learning, Webservice

## Abstract

Off-target drug interactions are a major reason for candidate failure in the drug discovery process. Anticipating potential drug’s adverse effects in the early stages is necessary to minimize health risks to patients, animal testing, and economical costs. With the constantly increasing size of virtual screening libraries, AI-driven methods can be exploited as first-tier screening tools to provide liability estimation for drug candidates. In this work we present ProfhEX, an AI-driven suite of 46 OECD-compliant machine learning models that can profile small molecules on 7 relevant liability groups: cardiovascular, central nervous system, gastrointestinal, endocrine, renal, pulmonary and immune system toxicities. Experimental affinity data was collected from public and commercial data sources. The entire chemical space comprised 289′202 activity data for a total of 210′116 unique compounds, spanning over 46 targets with dataset sizes ranging from 819 to 18896. Gradient boosting and random forest algorithms were initially employed and ensembled for the selection of a champion model. Models were validated according to the OECD principles, including robust internal (cross validation, bootstrap, y-scrambling) and external validation. Champion models achieved an average Pearson correlation coefficient of 0.84 (SD of 0.05), an R^2^ determination coefficient of 0.68 (SD = 0.1) and a root mean squared error of 0.69 (SD of 0.08). All liability groups showed good hit-detection power with an average enrichment factor at 5% of 13.1 (SD of 4.5) and AUC of 0.92 (SD of 0.05). Benchmarking against already existing tools demonstrated the predictive power of ProfhEX models for large-scale liability profiling. This platform will be further expanded with the inclusion of new targets and through complementary modelling approaches, such as structure and pharmacophore-based models. ProfhEX is freely accessible at the following address: https://profhex.exscalate.eu/.

**Supplementary Information:**

The online version contains supplementary material available at 10.1186/s13321-023-00728-6.

## Introduction

Nowadays, the concept of polypharmacology [1–4] predominates over the “one-target-one-disease” paradigm, thanks to a better understanding of drugs' mode of action and pathological processes. Polypharmacology opened various possibilities in drug discovery, related to repurposing and detecting potential off-target liabilities which can lead to adverse drug reactions [5]. Indeed, recent studies estimated that small molecule drugs bind on average 6–11 distinct off-targets excluding their intended pharmacological one [6, 7]. Off-target interactions are a major reason for drug candidate clinical failure and, eventually, post-market withdrawal [8–10]. It is necessary to anticipate such adverse effects at the very early stages of the drug discovery process to minimize health risks to patients, experimental animal testing, and economic costs [11]. The main reasons for failure are related to specific organ toxicities, with cardiovascular toxicity being the most common cause (17%), followed by hepatotoxicity (14%), renal toxicity (8%) and central nervous system toxicity (7%) [10]. The most notable example is the human voltage-gated potassium channel subfamily H member 2 (KCNH2, or hERG), which is linked to cardiac arrhythmias. Indeed, activity on hERG is a mandatory evaluation to be performed to meet regulatory requirements [11].

The constantly increasing size of virtual screening libraries limits the possibility to experimentally test drug candidates against a large panel of liability targets, even when employing in-vitro high-throughput screening (HTS) approaches. For this reason, AI-driven methods, which are already extensively employed in drug discovery for hits identification [12, 13], can be also exploited to provide liability annotations on the desired chemical space, driving towards the selection of safe candidates. Considering the importance of cardiotoxicity, neurotoxicity and hepatotoxicity in causing drug candidate failures [14–17], several silico models focusing on these endpoints have been published [18–20]. Pharmaceutical companies routinely explore screening molecules against tens of off-target [15, 17], which highlights the importance of polypharmacology during the drug discovery program. In this direction, a few SAR-based cheminformatics systems [21–23] have been developed to retrieve putative targets of a given compound by querying widely used databases such as ChEMBL or PubChem [24, 25]. However, these tools are not based on supervised learning algorithms but on simple searches by chemical similarity. The ToxCast and Tox21 [26, 27] programs contributed to generate a large chemical library of in-vitro HTS profiled compounds for a broad range of targets, including nuclear receptor and stress response signaling pathways. The COMPARA and CERAPP collaborative projects [28, 29] are two examples of first tier screening models built on HTS data for androgen and estrogen receptor activity, respectively. In the “big-data” domain, Lee et al. [30] generated supervised binary classification models on 1121 targets and 235 k compounds collected from CHEMBL. Mayr et al. [31] followed a similar approach, extending the number of compounds to 500 k. Arshadi et al. [32] adopted a complementary disease-related modelling task: hundreds of PubChem bioassays were mined with natural language processing techniques to assemble a series of modelling datasets relevant to key diseases (such as acute toxicity, cancer, infections, metabolism, etc.). Such models have the advantage of being able to directly provide the probability for a given compound to provoke unwanted effects on humans. On the other hand, building correct associations between targets and a given disease is the main source of uncertainty in this approach. Moreover, target-related models are needed if a target deconvolution analysis is envisaged.

A common limitation of currently published models is an overlap of the same public data sources (mainly ChEMBL and PubChem), which narrows data availability and makes them redundant in terms of applicability domain. Some considerations can also be done when the learning approach is simplified to a binary classification task [33–37], which can bring some drawbacks: (i) the possibility to rank molecules according to their affinity is lost; (ii) the training process becomes more complicated when the binning yields unbalanced datasets (iii) the determination of a predefined binning cutoff is difficult [34], as there could exist an intrinsic bias in the measured activity which is specific for each protein target. Finally, a comprehensive “compound’s liability profile” is rarely provided, as current systems output in a tabular format numerical predictions for each target, without any mechanistic connection to a given liability hazard.

To the best of our knowledge, no readily available screening platform provides a comprehensive mechanistically-driven liability profile. In this study, we aim to fill this gap with ProfhEX (AI-based liability profiler for small molecules in Exscalate), a suite of machine learning models hosted by the Exscalate computing center (https://www.exscalate.eu/en/platform.html) and freely accessible to the scientific community. In its first release, ProfhEX accounts for 46 Organization for Economic Co-operation and Development (OECD)-compliant [38] ligand-based models, built on a combined chemical space of 289′202 activity data for a total of 210′116 unique compounds. It provides a safety index regarding seven important liability profiles, such as cardiotoxicity, neurotoxicity, gastrointestinal, endocrine, pulmonary, renal, and immune system toxicities. We believe that ProfhEX would be a powerful first-tier virtual screening tool, providing researchers with useful information for drug discovery campaigns.

## Methods

**Fig. 1:**
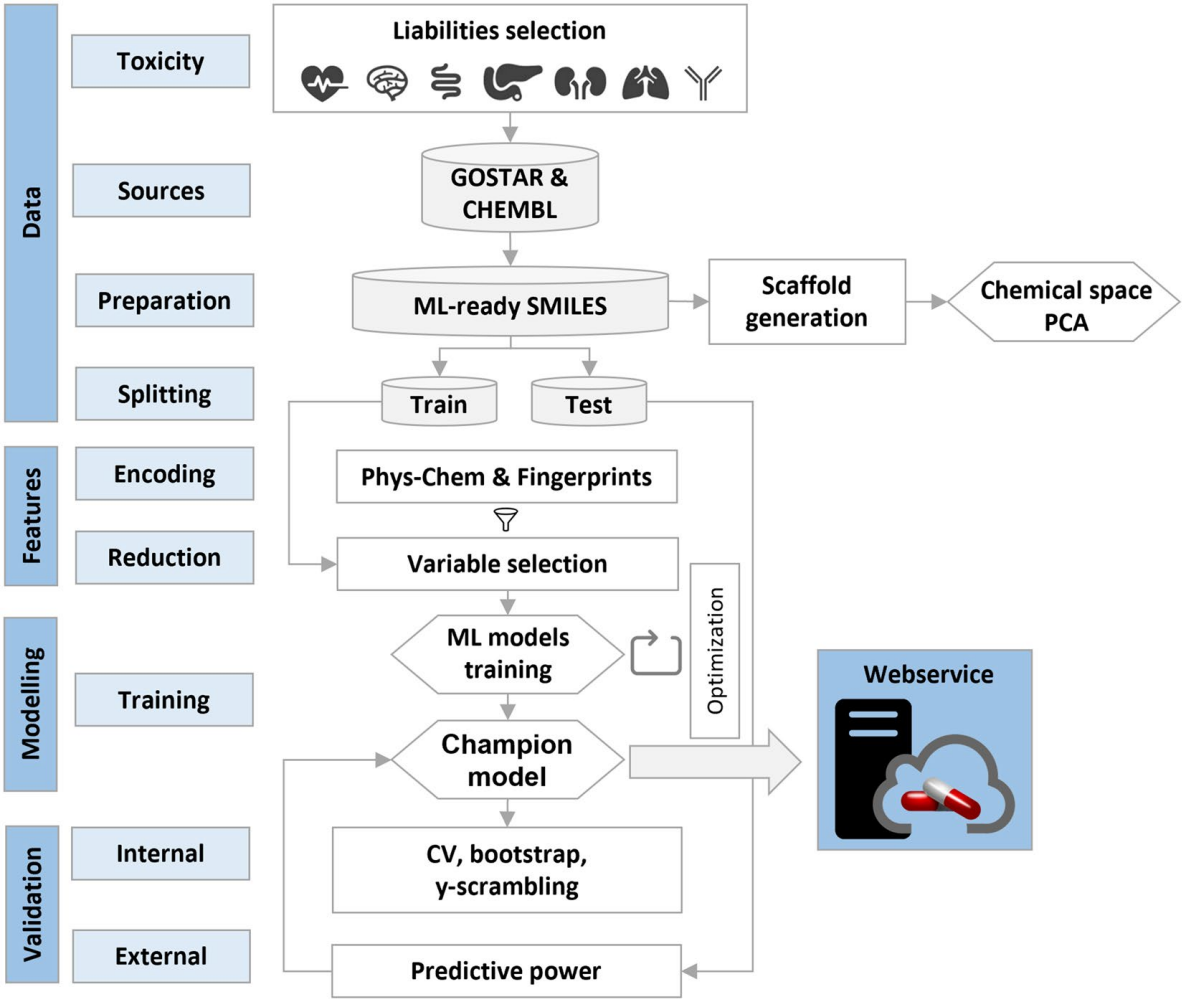
ProfhEX development workflow

Figure 1 depicts the development workflow of ProfhEX: (i) d*ata step*: selection of relevant targets for liability profiling [11], data collection from public and commercial data sources, and data preparation process; (ii) *features encoding step*: compounds encoding with physicochemical descriptors coupled with extended connectivity and feature invariant fingerprints; descriptor space reduction by feature selection techniques; (iii) *model generation step*: hyperparameter optimization, model training and champion model selection; *(iv) validation step*: internal validated by three complementary approaches and external validation on the test set partition; (v)* deployment*: webservice implementation.

### Data cleaning, feature encoding and dataset creation

Data preparation and feature encoding steps have been carried out in a Konstanz Information Miner [39] workflow. Activity data were collected from two sources: the publicly available ChEMBL database [24] and the commercial Excelra’s GOSTAR database (https://www.gostardb.com/), which is the world’s largest manually curated structure–activity-relationship database that collects comprehensive intelligence on bio-active compounds [12]. Activity data has been retrieved from both ChEMBL and GOSTAR in a similar way, as both databases have been designed with similar schemas. For the selected 46 targets all experimentally measured biological activity data were collected by UniProt identifier query. UniProt identifiers were retrieved from UniProt [40]. A series of sequential cleaning criteria have been applied to generate QSAR-ready entries. All measurements from sources other than “*homo sapiens*” or “*human*” have been excluded; censored values (i.e. > or <) have been excluded; only activity values encoded as “IC50”, “EC50”, “Ki or “Kd” have been considered and normalized to the negative log unit molar concentration (hereafter generally denoted as pACTIVITY), which is the dependent variable of the models. Despite functional (IC50/EC50) and binding (Ki/Kd) measurements refer to different biological activities, they are often combined for the development of first-tier screening models in order to maximize training set’s size and applicability domain [21, 22, 30, 34, 41, 42].

Compound’s structures originally available as SMILES strings were preprocessed and standardized in a Pipeline Pilot (v. 2018) protocol [43] by applying standard chemical compounds cleaning rules [44], such as removal of salts, standardization of functional groups (e.g. -nitro) and neutralization. Geometric optimization was not performed as employed descriptors are not conformer-dependent. De-duplication has been based on standardized SMILES matching. The median pACTIVITY value has been taken as a representative value when multiple pACTIVITY measurements were available for a given compound.

Feature encoding has been carried out using the RDKIT framework (https://www.rdkit.org/) available in KNIME (v. 4.5.2) [39]: 11 basic physicochemical properties coupled with extended connectivity (EC) and feature invariant (FC) fingerprints (radius of 6 and 1024 bits value each) have been generated, for a total of 2059 features. The value of 1024 was selected as an optimal fingerprint length to encode all datasets (from a few hundred up to several thousand compounds) without causing bit saturation. Finally, each dataset is partitioned into train/test set with an 80/20 ratio by stratified sampling (based on the dependent variable pACTIVITY). Stratified sampling has proven useful in evaluating performances when dealing with unbalanced class repartitioning. To verify the influence of train/test set composition, other methods such as random, diversity and scaffold-based sampling have been explored [42, 45, 46]. Hyperparameter optimization, training, and internal validation have been carried out on the train partition, whereas the test partition was used in external validation.

All datapoints collected from ChEMBL, the KNIME and Pipeline Pilot data preparation and modelling protocols and precalculated models, are freely available at the following Zenodo repository: https://doi.org/10.5281/zenodo.7665586.

### Chemical space analysis

Principal component analysis (PCA) is a dimensionality reduction method that allows visualizing with 2D plots multidimensional datasets [47]. Low variance (SD < 0.01) and highly correlated descriptors (R > 0.95) were excluded. PCA was not directly applied to individual compounds but to their “basic framework” scaffolds [48]. Starting from the widely accepted definition of scaffold (which is generated by removing all side chains and terminal atoms), a basic framework brings an even higher level of abstraction, having all the atoms converted to carbons but still maintaining features such as ramifications, double and aromatic bonds. Generated basic frameworks have been used as a grouping term of the initial chemical space, reducing the number of items from 210′116 unique compounds down to 39′247 scaffolds. In the group-by process, computed features have been averaged over the entries matching a given scaffold and standardized (i.e. autoscaled).

### Employed machine learning algorithms

All tasks related to feature selection, model training and scoring have been carried out with SAS Viya 3.5 software [49]. Tree based gradient boosting (GB) and random forest (RF) algorithms were employed for model generation. Gradient boosting [50] is a boosting approach that resamples the analysis data set several times to generate results that form a weighted average of the re-sampled data set. Tree-boosting creates a series of decision trees that are merged to form a single predictive model. Random forests [51] are a combination of tree predictors in which each tree depends on the values of a random vector sampled independently and with the same distribution for all trees in the forest. The performance of random forests is related to the quality of each tree in the forest. Because not all the trees “see” all the variables or observations, the trees of the forest tend to have a small correlation or no correlation. In addition, multi-linear regression (MLR) approach was implemented as baseline estimation.

### Autotune

A hybrid approach was used to automate the tuning phase of the model hyperparameters (SAS “AUTOTUNE procedure”). Briefly, in the first stage Latin Hypercube Sampling [52] is employed to generate a semi-random sample ensuring the uniqueness of the value-hyperparameter pair in all the experiments. The results of the first stage are used to initialize the evolutionary-inspired genetic algorithm optimization [53], which allows to efficiently explore the hyperparameters space. The tuning procedure has been run exclusively on the train partition of each dataset by fivefold cross validation split, and the root mean squared error was set as optimizing function to be minimized. This procedure was employed to tune the main parameters of GB and RF such as the number of trees, tree depths, number of bins, leaf size, learning rate and regularization L1 and L2. For each model, a time threshold of 100 min and a stagnation of the optimizing function over five sequential iterations were set as early stopping criteria.

### Model training and validation

The best set of hyperparameters from the autotune procedure was used for model training. Model robustness and predictive power have been evaluated using complementary approaches. For internal validation, fivefold cross validation procedure, 90/10% bootstrap sampling and y-randomization were used [54, 55]. All internal validation procedures were iterated 100 times. In contrast, the test set partition was employed for external validation, which never participated neither in model training nor in hyperparameter optimization. The RF or GB model with the highest Pearson coefficient was chosen as “champion” model for each considered target. This metric was chosen to avoid any potential bias caused by the markedly higher frequency of several specific values of the dependent variable. As seen in the pACTIVITY value distribution plot (Fig. 3), there is a noticeable number of experimental values in correspondence of key pACTIVITY -thresholds, such as 5 and 6 log units (corresponding to 10 and 1 µM, respectively). These values are frequently used in HTS single-concentration assays to discriminate potentially active compounds. This makes RMSE a less robust metric, whereas R coefficient seems to be a better indicator, being a normalized measurement of the covariance between experimental and predicted pACTIVITY.

### Applicability domain

Each QSAR model has its own applicability domain [54], which defines the chemical space boundaries inside which the relationship between structure and activity can be considered valid and therefore the model’s prediction reliable. A structure similarity approach has been employed to define the AD, which is similar to distance-based methods [56]. Structural similarity to training set’s compounds for a given prediction compound is estimated by the Tanimoto coefficient (Tc) computed on the 2048 fingerprint variables. Each model’s AD is considered fulfilled with Tc > 0.7. An overall AD score is assigned based on the fraction of models (out of the total 46) that had their individual AD fulfilled: higher values indicate a more reliable estimation.

### Evaluation metrics

Pearson correlation coefficient (R, Eq. 1), determination coefficient (R^2^, Eq. 2) and root mean squared error (RMSE, Eq. 3) were selected as the main metrics to monitor model performance. R coefficient has been calculated as:

1 $$R_{X,Y} \, = \,\frac{{cov\,\left( {X,\,Y} \right)}}{{s_{X} \,s_{Y} }}$$

where X indicates the actual values, Y the predicted values, $cov\left(X,Y\right)$ is the covariance ${s}_{X}$ and ${s}_{Y}$ are the standard deviation of X and Y, respectively.

R^2^ determination coefficient has been calculated as:

2 $$R^{2} \, = \,1\, - \,\frac{Rss}{{Tss}}$$

where R_ss_ and T_ss_ are the residual sum of squared and the total sum of squares, respectively.

RMSE has been calculated as:

3 $$RMSE{\mkern 1mu} = {\mkern 1mu} \sqrt {\sum\nolimits_{{i\, = \,1}}^{n} {\frac{{\left( {X_{i} {\mkern 1mu} - {\mkern 1mu} Y_{i} } \right)^{2} }}{n}} }$$

where X are the actual values, Y the predicted values and $n$ is the number of observations.

In addition, the enrichment factor (EF) and related ROC AUC were also computed. The EF at a given cutoff χ is calculated from the proportion of true active compounds in the selection set in relation to the proportion of true active compounds in the entire dataset (Eq. 3). To enable EF calculation, the top 2% of the pACTIVITY -sorted compounds for the given dataset have been labelled as true actives, whereas the remaining as inactives. We chose a variable cutoff rather than a fixed pACTIVITY value (for such analysis a pACTIVITY value between 5 and 6 is generally used [34]) for two reasons: (i) the datasets have different sizes and (ii) to have the same probability of randomly picking an active compound (i.e. the denominator of Eq. 3). The value of 2% has been chosen as a compromise between the minimum number of actives and dataset sizes. Furthermore, hit rate values between 1 and 5% are normally found in virtual screening benchmarking datasets [57].

The enrichment factor at different levels (1%, 5% and 10%) has been calculated as:

4 $$EF\, = \, {\raise0.7ex\hbox{${\frac{{A_{{{\chi }}} }}{{M_{{{\chi }}} }}}$} \!\mathord{\left/ {\vphantom {{\frac{{A_{{{\chi }}} }}{{M_{{{\chi }}} }}} \frac{A}{M}}}\right.\kern-0pt} \!\lower0.7ex\hbox{$\frac{A}{M}$}}$$

where χ is the top percentage in the distribution (assuming the values of 1, 5 and 10%), ${A}_{\upchi }$ is the number of active molecules in the top k% of the distribution, ${M}_{\upchi }$ is the number of molecules in the top k% of the distribution, A is the total number of actives and M is the total number of molecules.

## Results and discussion

### Target selections

The list of targets constituting ProfhEX has been taken from the study of Bowes et al. [11], where the Authors compiled a list of a “minimal panel of targets” that are routinely tested in-vitro for liability profiling by world-leading pharmaceutical companies. Relevant targets have been selected based on the probability of a hit on the target compared to the magnitude of the impact of this hit. For instance, hERG and muscarinic receptors are classified as high-rate/high-impact targets.

**Fig. 2:**
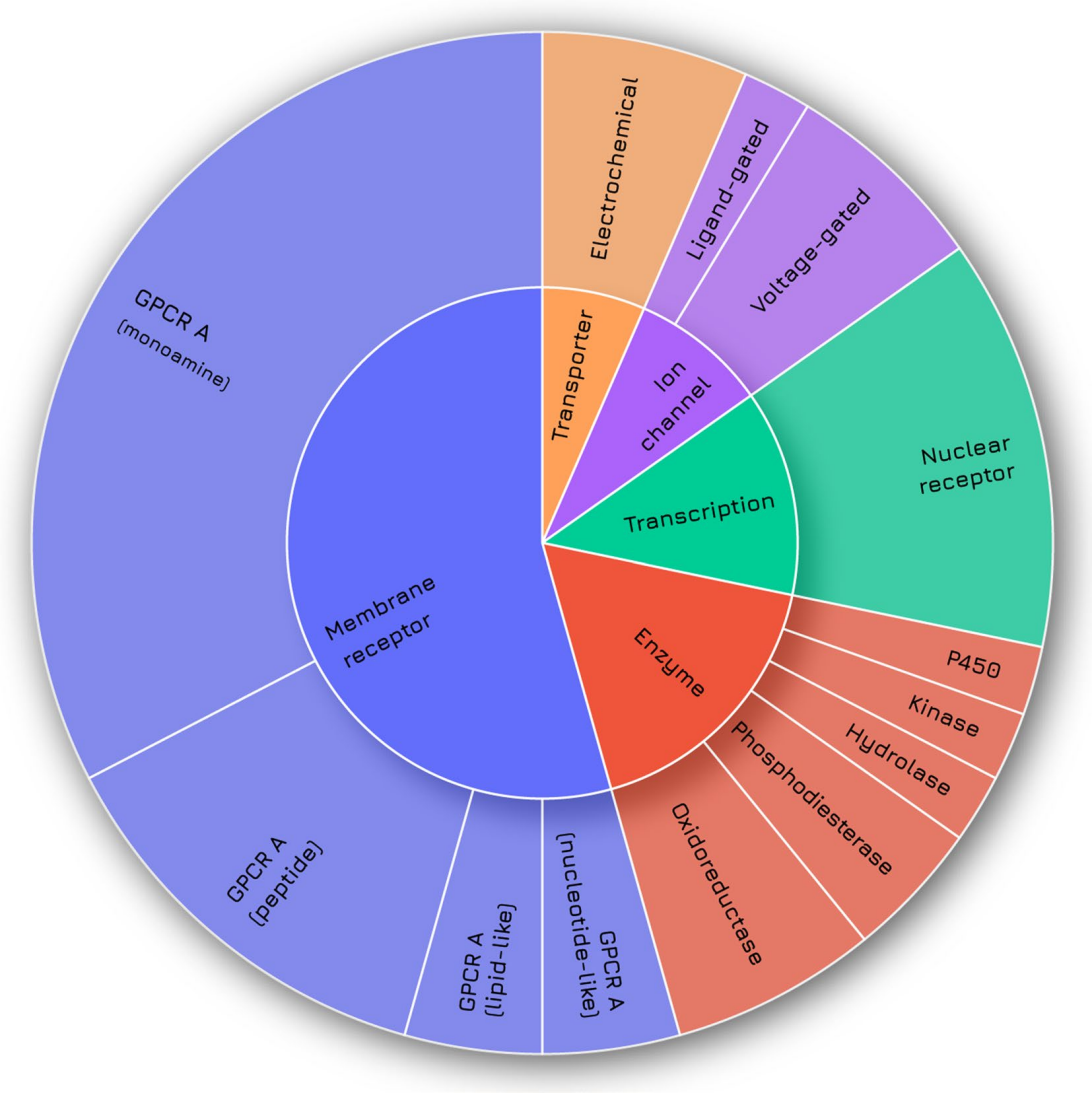
Protein family classification of the 46 selected targets

Table 1 reports the list of selected targets and Fig. 2 depicts their protein family classification: most of them are membrane receptors from the GPCR (G protein-coupled receptors) superfamily (25), followed by enzymes (8 members), transcription factors (6) ion channels (4) and transporters (3), for a total of 46 targets. Most of the selected targets are involved in the prediction of cardiovascular, central nervous system and gastrointestinal side effects. Additionally, several targets are relevant for more than one liability such as the dopamine receptors (DRD1 and DRD2), whose activation could lead to adverse cardiovascular, nervous, and immune system effects.

**Table 1:** Target-liability reference

Liability	Targets
CV (25)	ACHE, ADORA2A, ADRA1A, ADRA2A, ADRB2, AVPR1A, CHRM1, DRD1, DRD2, HRH1, HRH2, HTR1B, HTR2A, HTR2B, KCNH2, MAOA, OPRD1, OPRK1, OPRM1, PDE3A, PTGS2, SCN5A, SLC6A2, SLC6A4
CNS (19)	ADORA2A, ADRA1A, ADRA2A, CHRM1, CNR1, DRD1, DRD2, EDNRA, HTR1A, HTR1B, HTR2A, MAOA, OPRD1, OPRK1, OPRM1, PDE4D, SLC6A2, SLC6A3, SLC6A4
GI (13)	ACHE, ADRA1A, ADRB1, CCKAR, CHRM1, CHRM3, HRH2, HTR3A, OPRK1, OPRM1, PPARA, PPARD, PTGS1
ED (7)	AR, DRD2, EDNRA, ESR1, HTR1A, HTR3A, NR3C1
PU (5)	ACHE, ADRB2, CHRM3, HTR2B, PTGS1
RE (2)	AVPR1A, PTGS1
IM (6)	CNR2, HRH1, LCK, NR3C1, PDE4D, PTGS2

*CV* cardiovascular, *CNS* central nervous system, *GI* gastrointestinal, *ED* endocrine disruption, *PU* pulmonary, *RE* renal, *IM* immune. In brackets, the number of targets is reported

**Fig. 3:**
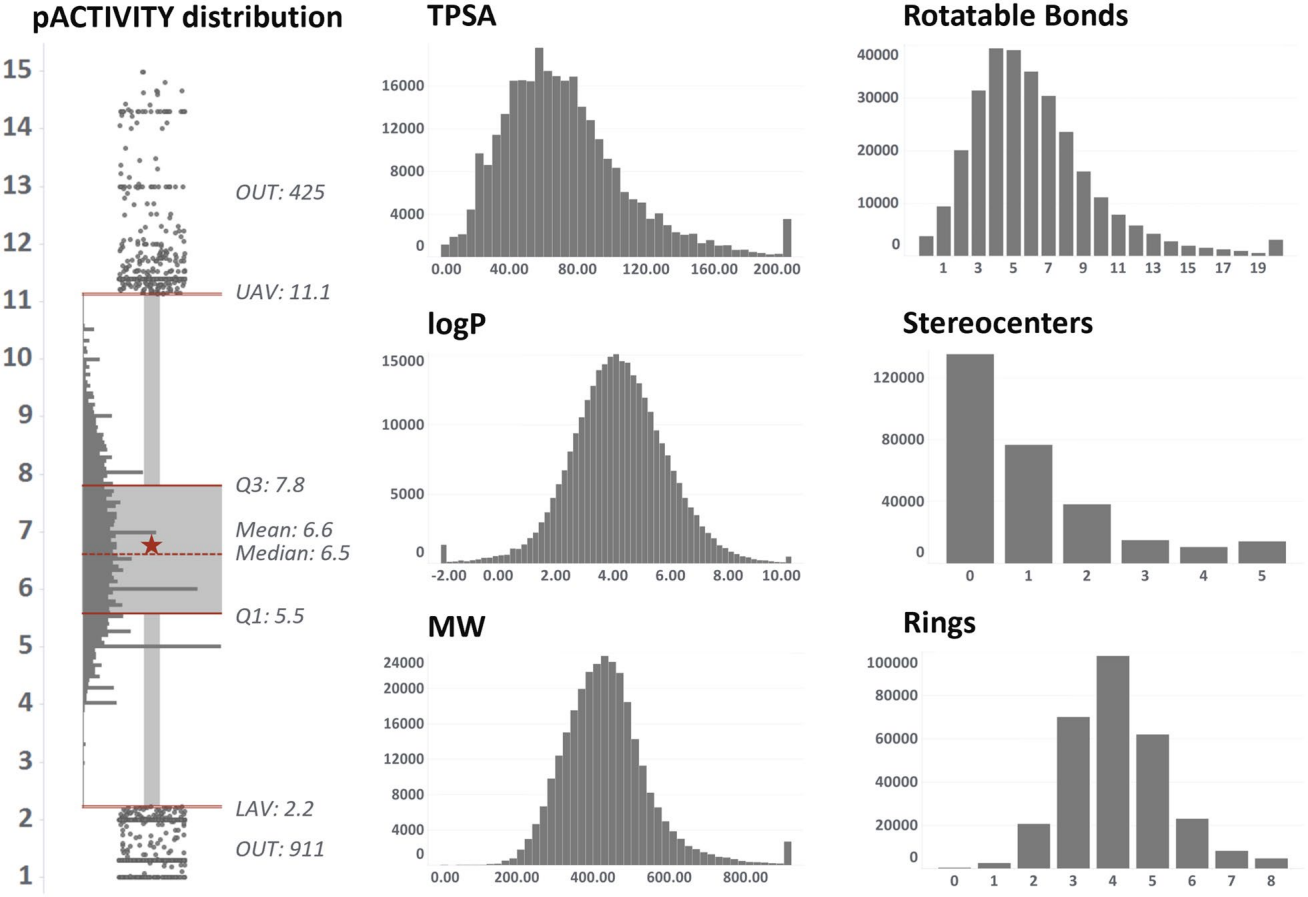
Key-properties distribution over the entire chemical space of the 46 considered targets. The boxplot depicts pACTIVITY values, whereas the histograms depict (in order from top-left to bottom-right), topological surface area, number of rotatable bonds, logP, number of stereocenters, molecular weight and number of aromatic rings

Dataset sizes range from 819 (HRH2—GPCR A histamine receptor) up to 18896 (CNR1—GPRC A cannabinoid receptor). The distribution of activity values and key-physicochemical properties over the entire chemical space is depicted in Fig. 3 (see Additional file 1: Table S1 for more details). Most of the basic properties exhibit a normal distribution with some degree of skewness, such as TPSA and number of rotatable bonds. The right tail of the distributions of MW, TPSA and number of rotatable bonds is populated by peptides and natural products, which normally have more branching and substituents than small molecules.

**Fig. 4:**
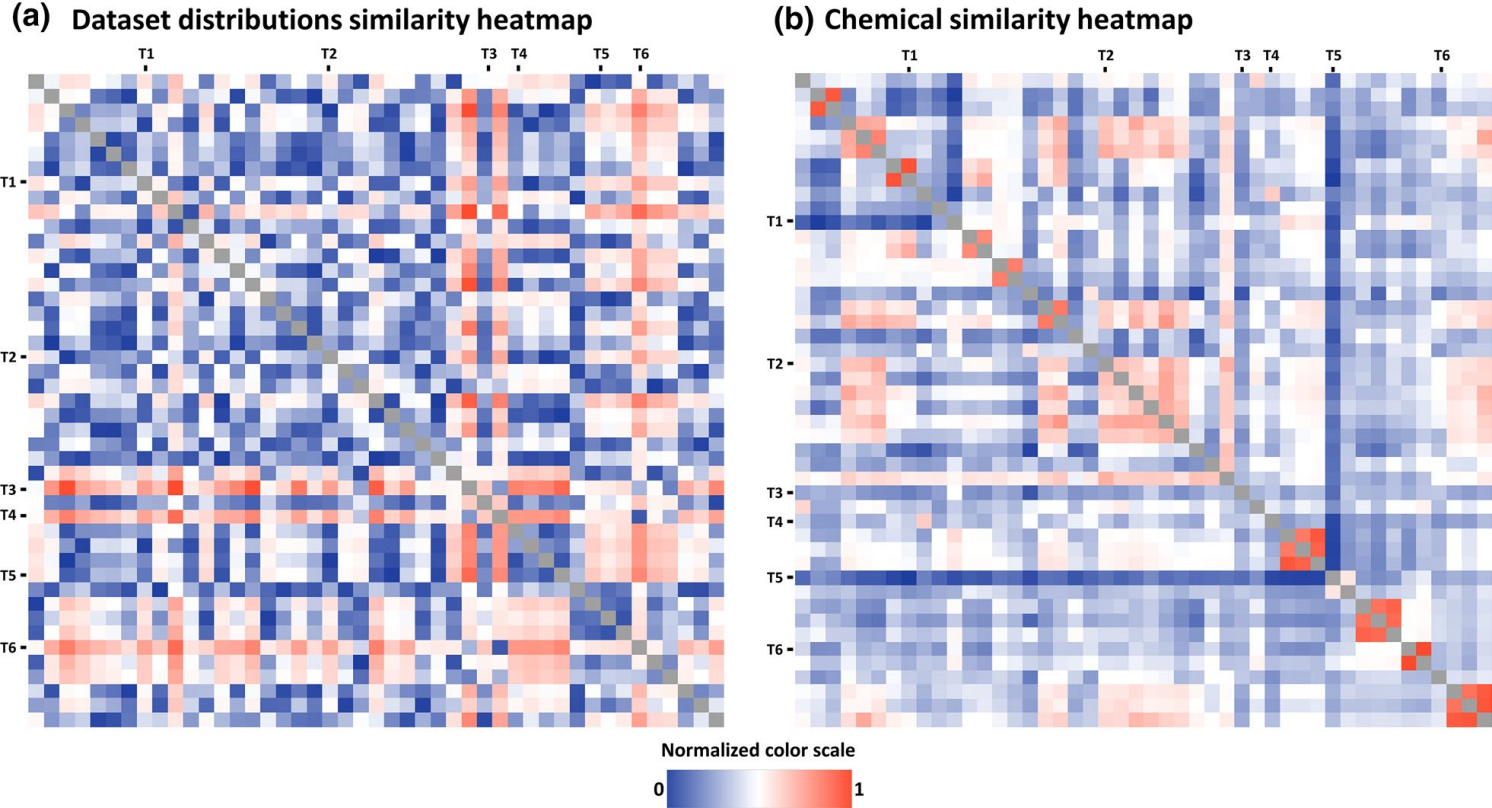
**a** Pairwise comparison of the 46 datasets pACTIVITY distributions computed by Kolmogorov–Smirnov statistical test. Analogously, **b**. depicts the average Tanimoto similarity coefficient among the compounds of the given datasets pair. The color scale has been normalized between 0 and 1: higher values (red) indicate that the given datasets pair show significantly different distributions **a** and higher chemical similarity **b**, as opposed to values closer to 0 (blue). Marked rows and columns with codes T1-6 highlight some targets referenced in the text, in order: ADRB1, ESR1, HTR1A, KNCH2, MAOA, PTGS1

The average pACTIVITY value over the 46 datasets ranges from 5.23 (MAOA) to 7.76 (AVPR1A), with an average of 6.6 log and SD of 0.6. This indicates a shift of individual pACTIVITY distribution mean values. The reasons for this deviation could be due to (i) an intrinsically higher receptor selectivity (i.e. it tends to be activated only by specific chemical families of small molecules); (ii) a bias of in in the design of experimentally tested chemical libraries. Concerning this last point, the ESR1, OPRM1 and ADRB2 datasets (high average pACTIVITY) come from patents and reviews describing the use of compounds for cancer, inflammation, osteoporosis and other disorders. On the other hand, the hERG, COX-1 and MAOA datasets (low average pACTIVITY) are mainly associated with liability studies aiming to demonstrate that the compounds are not active on high hazard/impact receptors.Fig. 4a (Additional file 1: Figure S3) illustrates the pairwise pACTIVITY distribution distances calculated from the Kolmogorov–Smirnov test [58]. Targets such as KNCH2 (T4), MAOA (T5) and PTGS1 (T6) have significantly different distributions (p < 0.05) compared to most of the targets, having the lowest average pACTIVITY values (around 5.5 log units). Figure 4b (Additional file 1: Figure S4) illustrates the pairwise average Tanimoto similarity between compounds in the dataset. Most of the time, datasets from the same protein family are clearly characterized by analogue chemical moieties, as in the case of ADRB1 (T1) with ADRB2 (Tanimoto = 0.91) and HTR1A (T2) with HTR1B HTR2A and HTR2B (Tanimoto ~ 0.48). On the other hand, the chemistry of tested compounds on PDE3A (T5) is noticeably different from all other datasets, except for PDE4A (Tanimoto = 0.37).

### Chemical space analysis

**Fig. 5:**
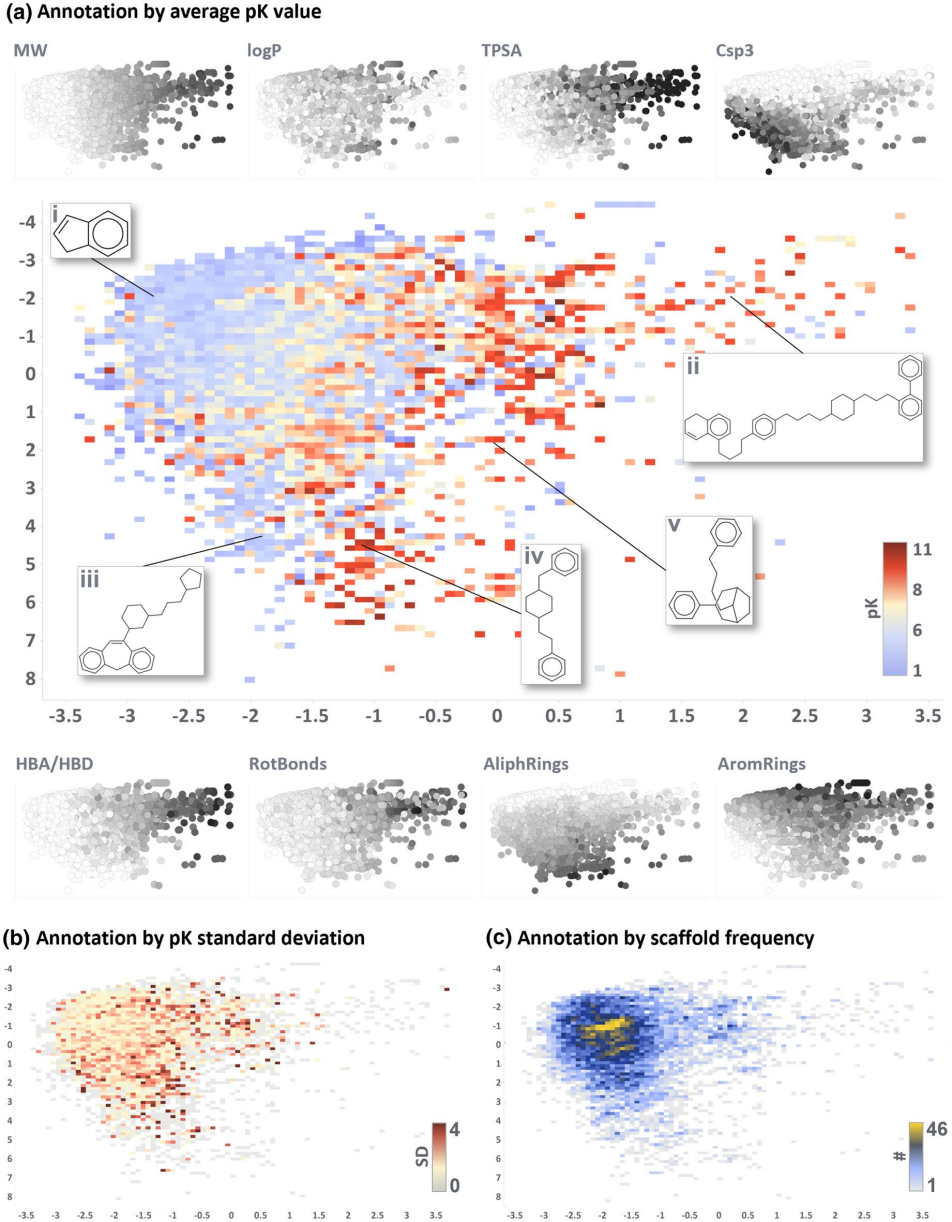
First two components of the principal component analysis of the 46 targets scaffold-based chemical space. Explained variance from the first two PC is 34%. Three heatmaps depict the chemical space annotated by the following properties: average pACTIVITY value **a**, pACTIVITY value standard deviation **b**, and scaffold occurrence over considered targets (**c**). Black and white scatterplots around the main plot **a** show the trend of key properties which participated in PCA (from lower to higher values for white and black, respectively). Some key scaffolds mentioned in the text have been highlighted (Latin numbers i-v)

Figure 5 depicts the PCA of the chemical space by means of annotated heatmaps. The cumulative variance explained by the first two components is 34% (28 and 6% respectively). Black and white scatterplots in Fig. 5a depict the trend of some key properties (see also loadings plot in Additional file 1: Figures S1 and S2). Molecular weight is one of the most influential features and distributes the scaffolds on the x-axis from lower (left) to higher (right) MW compounds. This trend has also been reported in other publications exploring chemical space comparison [59, 60]. Other properties such as topological surface area, number of aromatic rings, number of stereocenters and number of H-bond donor/acceptor, follow the same trend, which is quite expected as an increase in the molecular weight generally corresponds to a frequency increase of all chemotypes. The second principal component is mainly driven by the fraction of sp3 hybridized carbons and the number of aliphatic rings, both increasing when moving towards lower y-axis values (see Additional file 1: Figures S1 and S2).

Figure 5a is annotated by the average pACTIVITY value. Intuitively, smaller and common chemical moieties, such as *indane* (Fig. 5a, structure i), *benzene*, *naphthalene*, *cyclohexylbenzene* and *biphenyl* are the most frequent scaffolds, as they serve as common building blocks for more complex structures. Therefore, the average activity value over the entire chemical space is around 6 log units (Additional file 1: Table S1). Figure 5b, c annotate the chemical space by pACTIVITY standard deviation and scaffold frequency, respectively.

There is a clear gradient of increasing activity when shifting towards bigger and more peculiar scaffolds (e.g. Figure 5a structures ii–v): this is expected as such structures have been designed to be specifically selective against a given target (low standard deviation and frequency). For instance, the scaffold of the drug *haloperidol* (structure iv) appears in a total of 435 molecules with an average activity of 9.13 pACTIVITY (SD of 1.65), spanning over 14 different targets (e.g. adrenoreceptors, dopamine, histamine, serotonin, opioid and solute carrier receptors) [61–63]. The scaffold of the drug *fentanyl* (structure v) appears in 486 molecules, showing an average pACTIVITY of 7.1 (SD of 1.5) over 23 unique targets (e.g. ion channels, acetylcholinesterase, opioid, dopamine and cannabinoid receptors) [64–66]. On the other hand, some scaffolds are clearly inactive towards multiple targets, such as *dibenzo-oxazapine derivates* (structure iii) with an average pACTIVITY of 2.5 (SD of 1) over 5 unique targets (serotonin, histamine, dopamine and muscarinic receptors) [67].

### Models’ internal and external validation performance

**Fig. 6:**
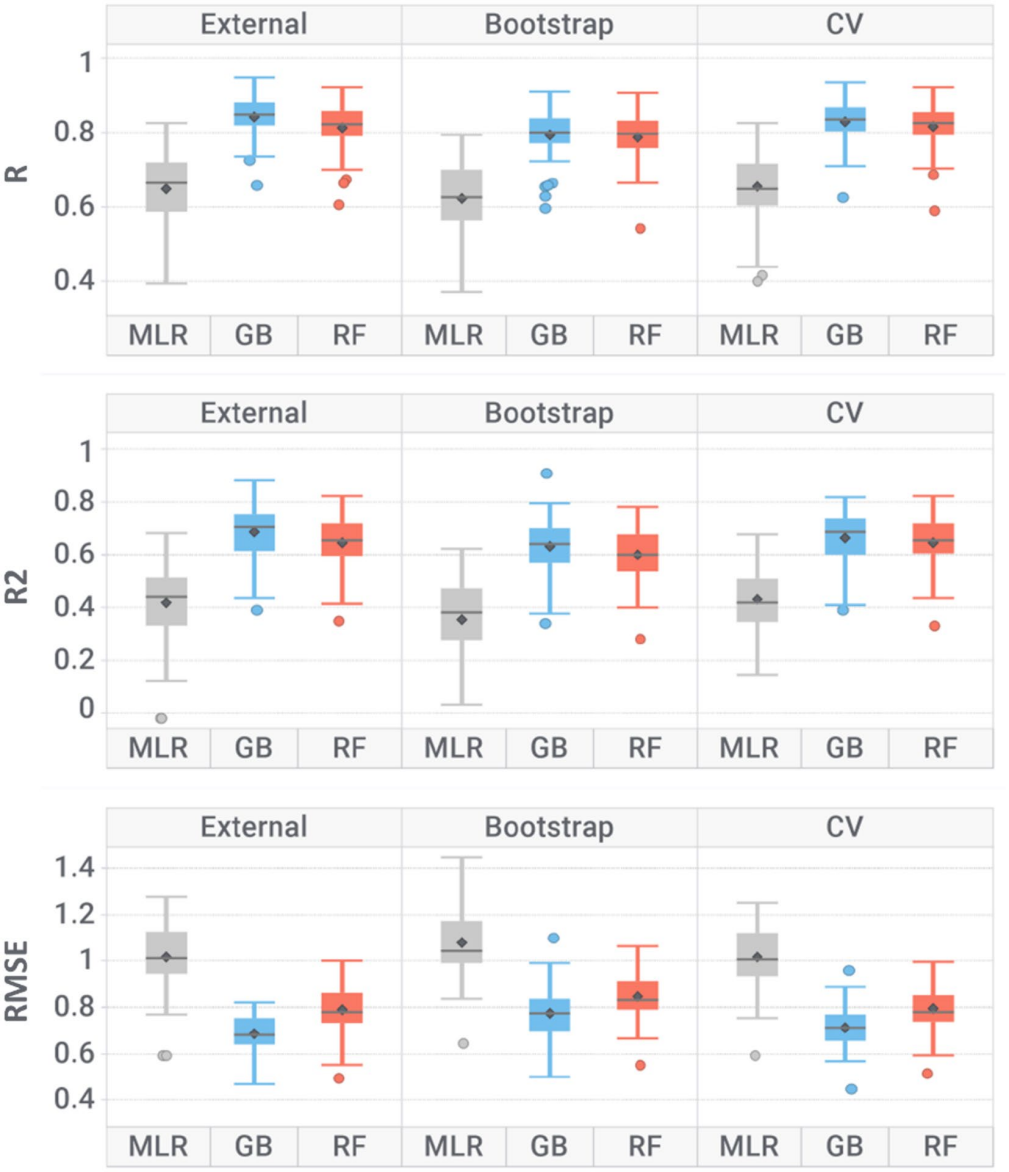
Box plot representation for determination coefficient (R^2^), Pearson correlation coefficient (R) and root mean squared error (RMSE) over the 46 modelled targets, grouped by algorithm and validation method. Where: *MLR* multi-linear regression, *GB* gradient boosting and *RF* random forest, *CV* cross validation

Table 2 and Fig. 6 report models performance averaged over the 46 modelled targets (see Additional file 1: Table S2 for individual target performance). In both internal and external validation, the most performing algorithm resulted to be GB (R = 0.79–0.84 and RMSE = 0.77–0.69), followed by RF (R = 0.79–0.81 and RMSE = 0.85–0.79) with slightly lower performance. GB has already been reported to outperform RF in modelling biological data [13, 68]. Due to the higher predictive power, GB-based models were designed as champions for all 46 targets. In fivefold cross validation, GB and RF scored R of 0.83 and 0.82; whereas in bootstrap 0.79 and 0.79, respectively. Similar performances in external validation (R of 0.84 and 0.81 for GB and RF, respectively) indicate that the models have good predictive power on unseen data and support the absence of overfitting. Train/test partitioning did not negatively affect model performances (Additional file 1: Figure S6, graphs a) and b)), as there is no significant difference (p > 0.05) in the model’s R^2^ and RMSE distributions. Moreover, R and R^2^ values for both GB and RF models are close to zero in y-scrambling simulations: such a drop in performance confirms that models are unlikely to be biased by chance correlations. Finally, significantly higher performances than the baseline MLR classifier indicate that GB and RF algorithms proved successful in learning meaningful structure–activity relationships for the considered datasets. Tree-based algorithms are very proficient in modelling toxicology data, as they are less prone to overfitting, less susceptible to outliers and not as heavily affected by noise as other algorithms [30, 69]. Toxicological in-vitro and in-vivo data is indeed highly affected by variability due to the high number of factors that contribute to error, such as experimental measurements, and inter-laboratories variability, lower accuracy of HTS methods and heterogeneous datasets composition (e.g. measurements coming from binding and functional assays) [70, 71]. The overall RMSE of trained models is 0.75 (SD = 0.09), which is comparable to the variability of experimental affinity measurements of 0.66 (SD = 0.22). Achieving a prediction error comparable to the experimental data variability support the validity of learned structure–activity relationships, as machine learning models cannot be more accurate than the error of training instances. When dealing with biological data, it has been reported that the variance in experimental measurements could contribute more to prediction error than the error from the model itself [69, 72, 73].

**Table 2:** Models’ performance averaged over the 46 targets for the given algorithm and validation approach

Validation	Algorithm	R	R^2^	RMSE
External	MLR	0.62 (0.1)	0.35 (0.2)	1.08 (0.17)
	GB	0.84 (0.05)	0.68 (0.1)	0.69 (0.08)
	RF	0.81 (0.07)	0.64 (0.11)	0.79 (0.1)
Bootstrap	MLR	0.66 (0.1)	0.43 (0.13)	1.02 (0.14)
	GB	0.79 (0.07)	0.63 (0.11)	0.77 (0.11)
	RF	0.79 (0.07)	0.6 (0.11)	0.85 (0.1)
fivefold cross validation	MLR	0.65 (0.11)	0.42 (0.15)	1.02 (0.14)
	GB	0.83 (0.06)	0.67 (0.1)	0.71 (0.09)
	RF	0.82 (0.06)	0.65 (0.1)	0.79 (0.1)
Y-scrambling	MLR	0.0 (0.1)	0.46 (0.14)	0.99 (0.13)
	GB	0.0 (0.02)	0.22 (0.09)	1.49 (0.28)
	RF	0.0 (0.02)	0.05 (0.01)	1.37 (0.26)

The standard deviation is reported in brackets

PTGS1 and PTGS2 were the lowest performing models (R = 0.6–0.66 and RMSE = 0.8–0.87), despite their relatively large size of roughly 3000 and 6300 compounds, respectively. One explanation could be related to their different properties compared to the other datasets (Fig. 4) which made the learning process more difficult.

We also verified the impact on model performances by merging different activity types (i.e. IC50 and Ki). First, the correlation between inhibition concentration and binding affinity has been quantified when multiple measurements were available for the same compound of a given dataset. On average, 225 compounds for each dataset had the double IC50/Ki annotation. The correlation is significant, with R = 0.73 (SD = 0.20, p < 0.05). This suggests that, as an approximation, IC50 and Ki measurements can be merged. To verify the effect on performances, original models (i.e. combined IC50 and Ki data) were retrained using KI and IC50 measurements only (Additional file 1: Table S3). As expected, the average training set size of these new models is much smaller, and not all targets could be efficiently modeled (e.g. PTGS1, LCK, PTGS2, PPARG, PPARD, HRH2, DRD1). We can see that the performance distributions (Additional file 1: Figure S5) are not significantly different, having an average R^2^ of 0.69 (SD = 0.10), 0.66 (SD = 0.10) and 0.71 (SD = 0.11) for combined, Ki-only and IC50-only models, respectively. This also applies to the models’ RMSE, being on average 0.70, 0.77, 0.66 for combined, Ki-only and IC50-only models, respectively. This means only a ± 10% range. This error is also comparable to the experimental variability of activity measurements: which is (on average across the datasets) 0.50 for Ki and 0.56 for IC50.

The Office of Economic Cooperation and Development (OECD) principles [26] for building robust quantitative structure–activity relationship models were followed. The five OECD principles are: (i) a defined endpoint; (ii) an unambiguous algorithm; (iii) a defined applicability domain; (iv) appropriate measures for goodness-of-fit, robustness, and predictivity; (v) and a mechanistic interpretation, if possible. In this study, the endpoint for each model is well defined and goodness-of-fit, robustness and predictivity were evaluated using internal (fivefold cross validation, bootstrap, y-scrambling) and external validation. The model’s applicability domain is evaluated by structural similarity comparison to the training set’s compounds.

### Models’ enrichment factor performance

**Fig. 7:**
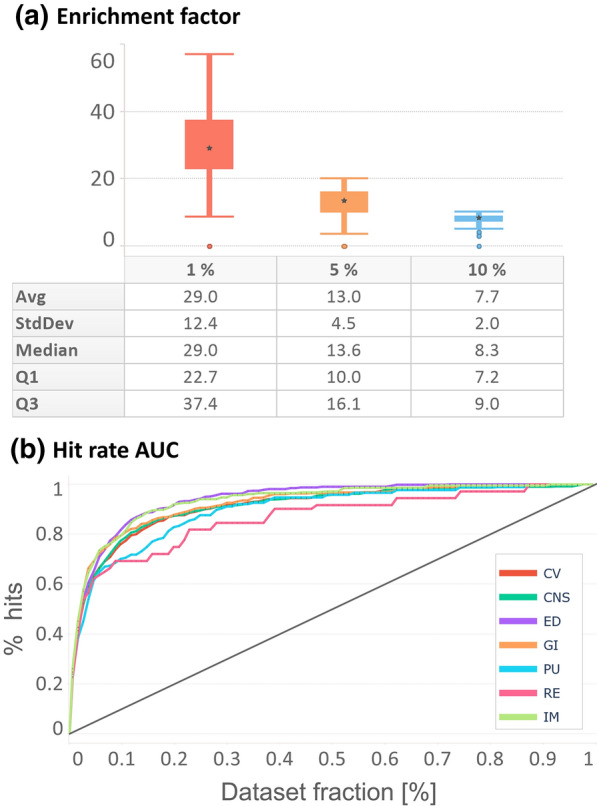
Enrichment factor and hit-detection performance for each liability group, **a** depicts a box plot representation of enrichment factor computed at 1, 5 and 10% cutoffs. **b** depicts normalized hit rate performance, where the x-axis represents the faction of the dataset and the y-axis the fraction of retrieved hits. Dataset size and the number of hits have been normalized across all the datasets to make them comparable

Figure 7a depicts the enrichment factor, whereas Fig. 7b the hit-detection performance in terms of AUC (Additional file 1: Table S2) grouped by liability group. All liability groups showed good hit-detection power with comparable performance, with the only exception of renal toxicity (RE), which showed lower discriminative power. However, this group is also composed of only two targets (PTGS1 and AVPR1A) which makes the evaluation less statistically robust. Overall, enrichment factor and AUC analysis showed that generated models can successfully retrieve active compounds at lower dataset faction levels, supporting their ability to discriminate true actives in large-volume virtual screening campaigns.

### Perspectives

Ligand-based approaches are generally easier to implement as they do not require knowledge of the crystal structure of the target protein, and thus can be trained by simpler 2D descriptors with good performances. Still, they possess some limitations: (i) the absence of target-related information inhibits the model to learn any rules related to protein–ligand interactions; (ii) the applicability domain is restricted to compounds that are similar to the chemical space delimited by their training set; and (iii) the distribution between active and inactive compounds is generally unbalanced in favor of the latter, leading to low recall rates and failure to reliably detect potential activity cliffs. To overcome these limitations, ligand-based or structure-based pharmacophore models can be developed to find common chemical features relevant to biological activity [74]. Recent applications of 3D pharmacophores reported their screening power in virtual screening studies and their synergistic combination with docking approaches [75]. Moreover, when the crystal structure is available, the inclusion of descriptors related to the crystal structure and docking simulations can be employed. All these approaches can be ensembled in consensus. Finally, to provide a comprehensive liability profile, it would be important to evaluate the metabolites of the query compounds, as they may provoke harmful responses once metabolized in the human body.

### ProfhEX webservice implementation

**Fig. 8:**
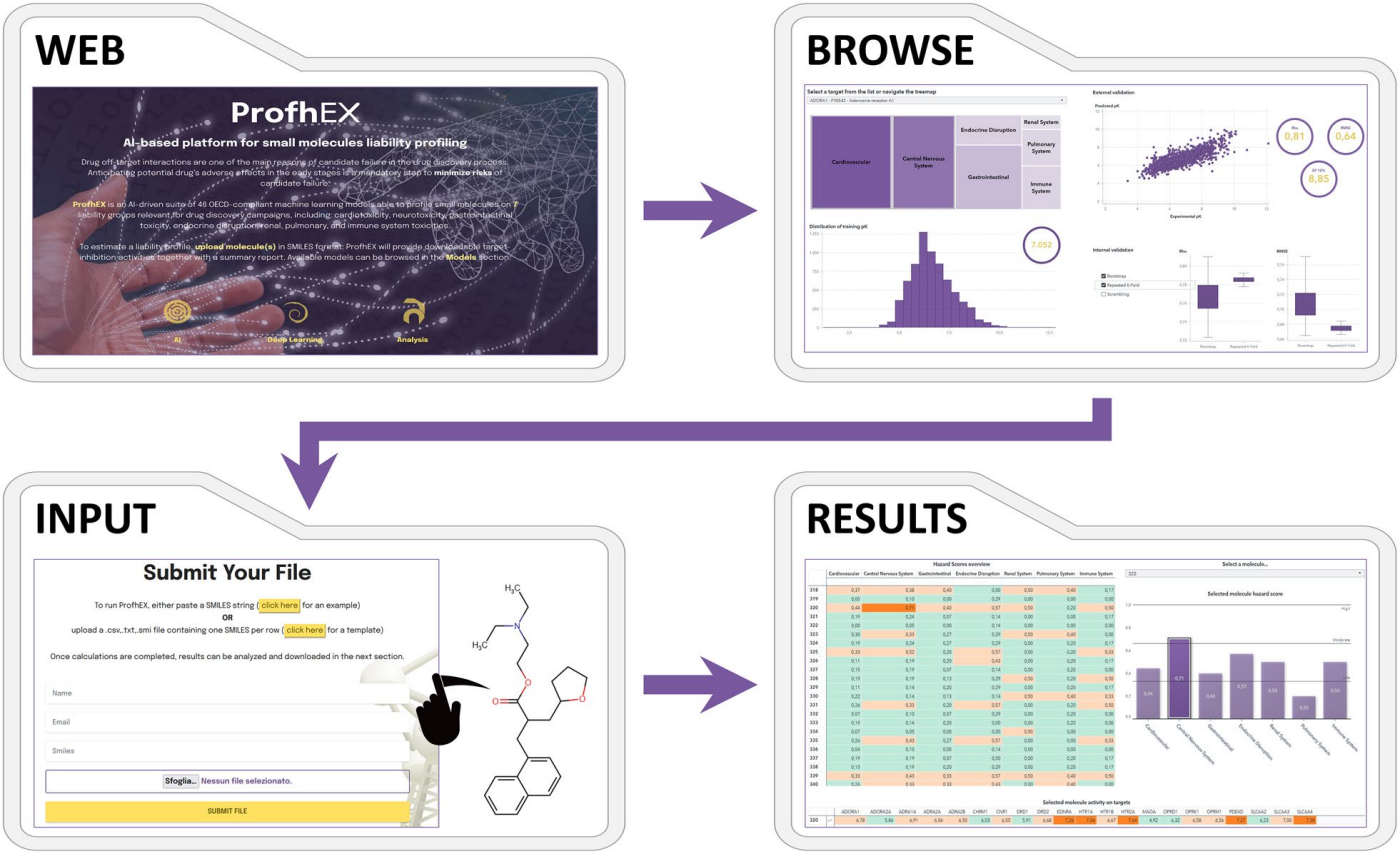
ProfhEX webservice workflow

Compounds should be submitted to ProfhEX (Fig. 8) via SMILES format. The prediction process leading to the generation of its liability profile comprises the following steps: (i) a vector of 46 predicted activities on the modelled targets is generated; (ii) predictions are binned into the two classes “concern” (C) and “not concern” (nC) based on a predefined pACTIVITY cutoff value of 6.5 (300 nM); (iii) classes are grouped into the 7 liability groups according to the liability mapping as described in Table 1; (iv) for each liability group a liability score is computed as the number of C labels out of the total number targets relevant for the given liability (Eq. 4).

5 $$Ls_{i} \, = \,\frac{{ C_{i} }}{{C_{i} \, + \,nC_{i} }}$$

where, ${Hs}_{i}$ is the liability score for the given liability group *i*, ranging from 0 (no target flagged as C) to 1 (all targets flagged as C); ${C}_{i}$ and ${nC}_{i}$ are the number of targets for the given liability group *i* flagged as C and nC, respectively.

A predefined threshold of 6.5 log units has been selected to achieve a balance between active and inactive compounds. A more generalizable approach would be to select a variable threshold depending on the distribution of experimental pACTIVITY measurements for the given target, for instance with the two-sigma rules. Such an approach could help in considering response biases present in the training datasets. Furthermore, a weighted approach could also be implemented when calculating the liability score, by putting more importance on key targets: for instance, voltage-gated channels such as KCNH2 (hERG) and KCNA5 (Kv1.5) are more relevant for cardiotoxicity than for the other liability groups.

### ProfhEX benchmarking

ProfhEX models were benchmarked against four works that performed large-scale modelling of affinity data: Cortés-Ciriano et al. [76], Yao et al. [77], Mayr et al. [31] and Bosc et al. [35]. We should underline that this is not a completely unbiased analysis due to the absence of a common benchmarking dataset. To mitigate this, we considered statistics originating from cross validation as they were always reported. Results are reported in Table 3 (extended statistics in Additional file 1: Table S4), and more details are provided in Additional file 1: Figures S7, S8 and S9.

**Table 3:** ProfhEX performances compared against already existing tools

Platform	No. targets	Type	Metric				
			RMSE	AUC	Sn*	Sp*	BA*
ProfhEX	46	R	0.71 (0.09)	0.91 (0.04)	0.87 (0.23)	0.97 (0.04)	0.92 (0.11)
Cortés-Ciriano et al. [76]	14	R	0.72 (0.07)	–	–	–	–
Yao et al. [77]	44	C	–	0.91 (0.04)	–	–	–
Mayr et al. [31]	29	C	–	0.84 (0.15)	–	–	–
Bosc et al. [35]*	27	C	–	–	0.75 (0.1)	0.93 (0.04)	0.85 (0.04)

No. targets indicate the number of targets in common with ProfhEX. Type “R” or “C” indicates the type of learning task, either regression or classification, respectively. Metrics Sn, Sp and BA stand for Sensitivity, Specificity and Balanced Accuracy, respectively. The standard deviation is reported in brackets

^*^Statistics come from a blind evaluation of the dataset provided by the authors

An evaluation of regression performances was only possible for 14 targets from Cortés-Ciriano et al. [76]. ProfhEX models are as performant as those reported by the authors, without a significant difference (p > 0.05) in the distribution of RMSE values (RMSE_ProfhEX_ = 0.71, RMSE_[76]_ = 0.72; Additional file 1: Figure S7). However, ProfhEX models perform noticeably better (18% lower error) for some targets, such as AR, LCK and SLC6A2. In Cortés-Ciriano et al. work all data from both human and non-human sources were retained, which could have increased data variability and model error. Performances in terms of ROC AUC (Additional file 1: Figure S8) were retrieved on all 46 targets from the works of Yao et al. [77] and Mayr et al. [31]. ProfhEX models (AUC_ProfhEX_ = 0.91) scored practically the same AUC as Yao et al. [77] (AUC_[77]_ = 0.91, p > 0.05) but a much better value than Mayr et al. [31] (AUC_[31]_ = 0.84, p < 0.05). Finally, we also performed a blind prediction on a dataset of affinity data taken from Bosc et al. [35]. After excluding compounds overlapping with ProfhEX training sets, the final number was 17580 (over 27 targets). The authors followed a less strict data-cleaning procedure, which explains a large number of new molecules. ProfhEX models scored very good performances in terms of balanced accuracy (BA), slightly better than those reported by Bosc et al. [35] (BA_ProfhEX_ = 0.92, BA_[35]_ = 0.85, p < 0.05).

As a case study, we carried out a blind evaluation on pre-registered, registered and withdrawn drugs, extracted from Cortellis Drug Discovery Intelligence (CDDI) database (https://access.cortellis.com/login?app=cortellis). After excluding compounds in ProfhEX datasets, 536 drugs were retained (Additional file 1: Table S5). Only a qualitative analysis through EF and AUC was possible, as drugs were annotated only by their primary target: 41/46 targets had at least one experimental annotation. ProfhEX models scored good performance in hit detection, with EFavg = 6.9 and AUCavg = 0.71 (Additional file 1: Figure S10, graphs (a) and (b)). Some EF spikes are caused by the limited number of annotated drugs for some targets. There is a drop in performance when using the liability groups scoring method we proposed (Eq. 4), with EFavg = 2.0 and AUCavg = 0.57 (Additional file 1: Figure S10, graphs (c) and (d)), suggesting that it may require further tuning depending on the use case.

## Conclusion

In this work we presented ProfhEX, an AI-driven web-based platform for small molecules liability profiling. In its first version, ProfhEX is composed of 46 OECD-compliant ligand-based machine learning models trained on binding affinity data, built on a combined dataset of 289′202 activity data for a total of 210′116 unique compounds. ProfhEX provides estimation for 7 important liability profiles, such as cardiovascular, central nervous system, gastrointestinal, endocrine disruption, renal, pulmonary and immune response toxicities.

Collected data from public and commercial databases was standardized and encoded by physicochemical descriptors and extended connectivity fingerprints. Gradient boosting and random forest algorithms were implemented. Models were validated according to the OECD principles, including robust internal (fivefold cross validation, bootstrap, y-scrambling) and external validation. The most performing model for each target was designed as a champion and implemented in ProfhEX. Champion models achieved an average Pearson correlation coefficient of 0.84 (SD of 0.05), an R^2^ determination coefficient of 0.68 (SD of 0.1) and a root mean squared error of 0.69 (SD of 0.08). All 7 liability profiles showed good hit-detection power with an average enrichment factor at 5% of 13.1 (SD of 4.5) and AUC of 0.92 (SD of 0.05). Performance comparison against already existing tools demonstrated the predictive power of ProfhEX models, supporting the platform’s utility for large-scale liability profiling. ProfhEX will be further expanded with the inclusion of new targets and by complementary modelling approaches, such as docking- and pharmacophore-based models.

All collected data from public sources together with the KNIME and Pipeline Pilot protocols are available at the following Zenodo repository: https://doi.org/10.5281/zenodo.7665586. ProfhEX is freely accessible at the following address: https://profhex.exscalate.eu/.

## Supplementary Information

**Additional file 1: Table S1.** Detailed statistics on the selected 46 targets, listing their Uniprot ID, the number of unique molecules, the average pK value (range in brackets), pK standard deviation and for which liabilities the target is relevant for. **Table S2.** Detailed performance of generated models. The Pearson correlation coefficient (R), the R2 determination coefficient and the Root Mean Squared Error (RMSE), are reported for each type of validation: validation set (external), fivefold cross validation (CV), bootstrap and y-scrambling. **Figure S1.** PCA loadings plot. **Figure S2.** PCA explained variance plot. **Figure S3.** Extended version of Fig. 4-a. Pairwise comparison of the 46 datasets pACTIVTY distributions computed by Kolmogorov–Smirnov statistical test. The color scale has been normalized between 0 and 1: higher values (red) indicate that the given datasets pair show significantly different distributions, as opposite to values closer to 0 (blue). **Figure S4.** Extended version of Fig. 4-b. Pairwise average Tanimoto similarity among the compounds of the given datasets pair. The color scale has been normalized between 0 and 1: higher values (red) indicate that the given datasets pair show higher chemical similarity, as opposite to values closer to 0 (blue). **Table S3.** Dataset size, IC50/Ki measurements correlation and performance comparison between Combined, IC50-only and Ki-only models. **Figure S5.** Plot of R2 performances of Table S3. **Figure S6.** External validation performance in terms of R2 (a) and RMSE (b) according to different train/test splitting strategies. Stratified bar (grey) is referred to R2_ext_ in table S2. **Table S4.** ProfhEX benchmarking against already published models. **Figure S7.** Comparison of ProfhEX performances (in terms of cross-validated RMSE) against the models published in the work Cortes-Ciriano [76]. **Figure S8.** Comparison of ProfhEX performances (in terms of cross-validated ROC AUC) against the models published in the works of Yao et al. [77] and Mayr et al. [31]. **Figure S9.** Comparison of ProfhEX performances (in terms of balanced accuracy) against the blind dataset selected from Bosc et al. [35]. **Figure S10.** Enrichment factor performance on the CDDI database. Plots a) and b) depict the enrichment factor and AUC based on liability group scores; whereas c) and d) depict the enrichment factor and AUC for the individual targets. EF has been computed at 1, 5, 10% cutoffs. **Table S5.** List of 536 pre-registered, registered and withdrawn drugs taken from Cortellis Drug Discovery Intelligence database.

## Data Availability

The dataset supporting the conclusions of this article is available via Zenodo repository at https://doi.org/10.5281/zenodo.7665586.

## Abbreviations

AD: Applicability domain
CNS: Central nervous system
CV: Cardiovascular
ED: Endocrine disruption
EF: Enrichment factor
GB: Gradient boosting
GI: Gastrointestinal
HTS: High-throughput screening
IM: Immune
ML: Machine learning
MLR: Multiple linear regression
OECD: Organization for economic co-operation and development
PCA: Principal component analysis
PU: Pulmonary
R: Pearson correlation coefficient
R^2^: Determination coefficient
RE: Renal
RF: Random forest
RMSE: Root mean squared error
SD: Standard deviation
