# Supplementary material for: ProfhEX: AI-based platform for small molecules liability profiling
Source: J Cheminform. 2023 Jun 9;15:60. doi: 10.1186/s13321-023-00728-6 (PMC10251600; doi:10.1186/s13321-023-00728-6)
Supplement: Supplementary file 1 — Additional file 1: Table S1. Detailed statistics on the selected 46 targets, listing their Uniprot ID, the number of unique molecules, the average pK value (range in brackets), pK standard deviation and for which liabilities the target is relevant for. Table S2. Detailed performance of generated models. The Pearson correlation coefficient (R), the R2 determination coefficient and the Root Mean Squared Error (RMSE), are reported for each type of validation: validation set (external), fivefold cross validation (CV), bootstrap and y-scrambling. Figure S1. PCA loadings plot. Figure S2. PCA explained variance plot. Figure S3. Extended version of Fig. 4-a. Pairwise comparison of the 46 datasets pACTIVTY distributions computed by Kolmogorov–Smirnov statistical test. The color scale has been normalized between 0 and 1: higher values (red) indicate that the given datasets pair show significantly different distributions, as opposite to values closer to 0 (blue). Figure S4. Extended version of Fig. 4-b. Pairwise average Tanimoto similarity among the compounds of the given datasets pair. The color scale has been normalized between 0 and 1: higher values (red) indicate that the given datasets pair show higher chemical similarity, as opposite to values closer to 0 (blue). Table S3. Dataset size, IC50/Ki measurements correlation and performance comparison between Combined, IC50-only and Ki-only models. Figure S5. Plot of R2 performances of Table S3. Figure S6. External validation performance in terms of R2 (a) and RMSE (b) according to different train/test splitting strategies. Stratified bar (grey) is referred to R2ext in table S2. Table S4. ProfhEX benchmarking against already published models. Figure S7. Comparison of ProfhEX performances (in terms of cross-validated RMSE) against the models published in the work Cortes-Ciriano [76]. Figure S8. Comparison of ProfhEX performances (in terms of cross-validated ROC AUC) against the models published in the works of Yao et al. [77] a [file 13321_2023_728_MOESM1_ESM.docx]

**ProfhEX: AI-based platform for small molecules liability profiling**

Filippo Lunghini^1^, Anna Fava^1^, Vincenzo Pisapia^2^, Francesco Sacco^2^, Daniela Iaconis^1^ and Andrea Rosario Beccari^1,*^

^1^EXSCALATE, Dompé Farmaceutici SpA, Via Tommaso de Amicis 95, Napoli 80123, Italy

^2^SAS Institute, Professional Service Department, Via Darwin 20/22, Milano 20143, Italy

KEYWORDS: virtual screening, liability profiling, polypharmacology, machine learning, webservice

**Additional file**

**Table S1.** Detailed statistics on the selected 46 targets, listing their Uniprot ID, the number of unique molecules, the average pK value (range in brackets), pK standard deviation and for which liabilities the target is relevant for.

| # | Protein family | Uniprot | Name | # mols | pK_average_ (min - max) | SD | Liabilities |
| --- | --- | --- | --- | --- | --- | --- | --- |
| 1 | Electrochemical | P23975 | SLC6A2 | 7398 | 6.78 (1.8 - 10.2) | 1.24 | CV, CNS |
| 2 | Electrochemical | Q01959 | SLC6A3 | 4658 | 6.47 (1.0 - 10.8) | 1.15 | CNS |
| 3 | Electrochemical | P31645 | SLC6A4 | 11059 | 6.93 (1.2 - 14.4) | 1.33 | CV, CNS |
| 4 | GPCR A (monoamine) | P35348 | ADRA1A | 2898 | 7.49 (2.4 - 11.0) | 1.31 | CV, CNS, GI |
| 5 | GPCR A (monoamine) | P08913 | ADRA2A | 3513 | 6.86 (1.4 - 11.3) | 1.17 | CV, CNS |
| 6 | GPCR A (monoamine) | P18089 | ADRA2B | 2168 | 6.83 (1.4 - 10.0) | 1.17 | CV, CNS |
| 7 | GPCR A (monoamine) | P08588 | ADRB1 | 2240 | 6.61 (3.1 - 10.7) | 1.34 | CV, GI |
| 8 | GPCR A (monoamine) | P07550 | ADRB2 | 4025 | 7.53 (1.0 - 12.1) | 1.51 | CV, PU |
| 9 | GPCR A (monoamine) | P11229 | CHRM1 | 7576 | 6.28 (1.0 - 15.0) | 1.25 | CV, CNS, GI |
| 10 | GPCR A (monoamine) | P20309 | CHRM3 | 6886 | 7.12 (1.0 - 15.0) | 1.91 | GI, PU |
| 11 | GPCR A (monoamine) | P21728 | DRD1 | 2293 | 6.51 (3.0 - 10.3) | 1.07 | CV, CNS |
| 12 | GPCR A (monoamine) | P14416 | DRD2 | 13497 | 6.77 (1.0 - 11.1) | 1.2 | CV, CNS, ED |
| 13 | GPCR A (monoamine) | P35367 | HRH1 | 3347 | 6.44 (2.0 - 13.0) | 1.37 | CV, IM |
| 14 | GPCR A (monoamine) | P25021 | HRH2 | 819 | 5.93 (3.0 - 13.0) | 1.19 | CV, GI |
| 15 | GPCR A (monoamine) | P08908 | HTR1A | 9021 | 7.3 (1.0 - 13.7) | 1.32 | CNS, ED |
| 16 | GPCR A (monoamine) | P28222 | HTR1B | 1540 | 7.12 (4.0 - 10.1) | 1.3 | CV, CNS |
| 17 | GPCR A (monoamine) | P28223 | HTR2A | 11716 | 6.7 (1.3 - 12.5) | 1.62 | CV, CNS |
| 18 | GPCR A (monoamine) | P41595 | HTR2B | 3283 | 6.18 (1.3 - 11.0) | 1.7 | CNS, ED, PU |
| 19 | GPCR A (nucleotide-like) | P30542 | ADORA1 | 9009 | 6.64 (3.2 - 12.8) | 1.13 | CV, CNS |
| 20 | GPCR A (nucleotide-like) | P29274 | ADORA2A | 15021 | 7.04 (1.9 - 13.0) | 1.36 | CV, CNS |
| 21 | GPCR A (peptide) | P37288 | AVPR1A | 5878 | 7.76 (3.1 - 11.4) | 1.18 | CV, RE |
| 22 | GPCR A (peptide) | P32238 | CCKAR | 941 | 7.23 (1.7 - 11.6) | 1.7 | GI |
| 23 | GPCR A (peptide) | P21554 | CNR1 | 18896 | 6.69 (1.0 - 12.5) | 1.47 | CNS |
| 24 | GPCR A (peptide) | P34972 | CNR2 | 18442 | 7.05 (1.8 - 12.1) | 1.36 | IM |
| 25 | GPCR A (peptide) | P25101 | EDNRA | 4198 | 6.6 (1.8 - 12.5) | 1.92 | CNS, ED |
| 26 | GPCR A (peptide) | P41143 | OPRD1 | 7993 | 7.39 (2.1 - 10.8) | 1.84 | CV, CNS |
| 27 | GPCR A (peptide) | P41145 | OPRK1 | 9073 | 7.11 (2.0 - 12.3) | 1.55 | CV, CNS, GI |
| 28 | GPCR A (peptide) | P35372 | OPRM1 | 13379 | 7.28 (1.7 - 13.4) | 1.69 | CV, CNS, GI |
| 29 | Hydrolase | P22303 | ACHE | 4759 | 6.1 (1.2 - 14.3) | 1.63 | CV, GI, PU |
| 30 | Kinase | P06239 | LCK | 3084 | 6.67 (3.3 - 11.0) | 1.43 | IM |
| 31 | Ligand-gated | P46098 | HTR3A | 853 | 7.3 (1.5 - 10.4) | 1.55 | GI, ED |
| 32 | Nuclear receptor | P10275 | AR | 6585 | 6.53 (1.4 - 12.1) | 1.23 | ED |
| 33 | Nuclear receptor | P03372 | ESR1 | 6659 | 7.38 (1.7 - 14.1) | 1.78 | ED |
| 34 | Nuclear receptor | P04150 | NR3C1 | 10030 | 7.07 (3.5 - 12.2) | 1.55 | ED, IM |
| 35 | Nuclear receptor | Q07869 | PPARA | 6967 | 6.03 (1.6 - 13.3) | 1.27 | GI |
| 36 | Nuclear receptor | Q03181 | PPARD | 4992 | 6.15 (2.7 - 12.0) | 1.32 | GI |
| 37 | Nuclear receptor | P37231 | PPARG | 7567 | 6.17 (1.4 - 13.0) | 1.21 | GI |
| 38 | Oxidoreductase | P21397 | MAOA | 3897 | 5.23 (1.4 - 12.3) | 1.11 | CV, CNS |
| 39 | Oxidoreductase | P23219 | PTGS1 | 2995 | 5.26 (1.4 - 11.5) | 1.08 | GI, PU, RE |
| 40 | Oxidoreductase | P35354 | PTGS2 | 6384 | 6.05 (2.2 - 11.7) | 1.22 | CV, IM |
| 41 | P450 | P11511 | CYP19A1 | 3728 | 6.27 (1.6 - 11.4) | 1.44 | GI |
| 42 | Phosphodiesterase | Q14432 | PDE3A | 976 | 6.5 (2.0 - 11.0) | 1.22 | CV |
| 43 | Phosphodiesterase | Q08499 | PDE4D | 2710 | 5.87 (2.0 - 11.0) | 1.77 | CNS, IM |
| 44 | Voltage-gated | P22460 | KCNA5 | 2135 | 5.97 (2.7 - 8.3) | 0.77 | CV |
| 45 | Voltage-gated | Q12809 | KCNH2 | 11325 | 5.44 (1.0 - 9.9) | 0.95 | CV |
| 46 | Voltage-gated | Q14524 | SCN5A | 2789 | 5.65 (1.1 - 9.6) | 1.31 | CV |

**Table S2.** Detailed performance of generated models. The Pearson correlation coefficient (R), the R2 determination coefficient and the Root Mean Squared Error (RMSE), are reported for each type of validation: validation set (external), 5-fold cross validation (CV), bootstrap and y-scrambling.

| # | Target | R | | | | R2 | | | | RMSE | | | | EF | | | AUC |
| --- | --- | --- | --- | --- | --- | --- | --- | --- | --- | --- | --- | --- | --- | --- | --- | --- | --- |
|  |  | ***Ext.*** | ***CV*** | ***Boot.*** | ***Y.*** | ***Ext.*** | ***CV*** | ***Boot.*** | ***Y.*** | ***Ext.*** | ***CV*** | ***Boot.*** | ***Y.*** | **1%** | **5%** | **10%** |  |
| 1 | ACHE | 0.84 | 0.85 | 0.81 | 0,00 | 0.64 | 0.65 | 0.59 | 0,00 | 0.82 | 0.84 | 0.96 | 1,65 | 40.5 | 13.7 | 8.4 | 0.97 |
| 2 | ADORA1 | 0.81 | 0.79 | 0.77 | 0,01 | 0.56 | 0.53 | 0.48 | 0,00 | 0.64 | 0.67 | 0.71 | 1,14 | 41.1 | 14.4 | 8.8 | 0.95 |
| 3 | ADORA2A | 0.86 | 0.85 | 0.82 | 0,00 | 0.67 | 0.66 | 0.6 | 0,00 | 0.65 | 0.67 | 0.76 | 1,30 | 16.6 | 8.0 | 6.0 | 0.91 |
| 4 | ADRA1A | 0.8 | 0.79 | 0.75 | 0,00 | 0.69 | 0.61 | 0.62 | 0,00 | 0.77 | 0.8 | 0.85 | 1,37 | 32.3 | 13.4 | 7.5 | 0.94 |
| 5 | ADRA2A | 0.82 | 0.81 | 0.78 | 0,00 | 0.61 | 0.6 | 0.59 | 0,00 | 0.64 | 0.66 | 0.72 | 1,18 | 28.4 | 16.1 | 8.6 | 0.92 |
| 6 | ADRA2B | 0.87 | 0.83 | 0.78 | 0,00 | 0.61 | 0.62 | 0.51 | 0,01 | 0.57 | 0.58 | 0.68 | 1,13 | 52.7 | 19.8 | 10.1 | 0.95 |
| 7 | ADRB1 | 0.83 | 0.84 | 0.78 | -0,01 | 0.69 | 0.69 | 0.69 | 0,00 | 0.72 | 0.7 | 0.77 | 1,37 | 35.9 | 17.4 | 8.9 | 0.90 |
| 8 | ADRB2 | 0.88 | 0.87 | 0.84 | -0,01 | 0.74 | 0.71 | 0.68 | 0,00 | 0.71 | 0.75 | 0.83 | 1,54 | 18.8 | 9.3 | 7.4 | 0.92 |
| 9 | AR | 0.87 | 0.86 | 0.82 | 0,00 | 0.69 | 0.69 | 0.65 | 0,00 | 0.6 | 0.61 | 0.66 | 1,24 | 37.9 | 12.7 | 7.0 | 0.92 |
| 10 | AVPR1A | 0.86 | 0.84 | 0.81 | -0,01 | 0.73 | 0.66 | 0.67 | 0,00 | 0.62 | 0.66 | 0.73 | 1,25 | 29.2 | 14.7 | 7.8 | 0.96 |
| 11 | CCKAR | 0.89 | 0.89 | 0.84 | 0,01 | 0.8 | 0.82 | 0.91 | 0,00 | 0.71 | 0.72 | 0.85 | 1,70 | 0.0 | 0.0 | 0.0 | 0.91 |
| 12 | CHRM1 | 0.85 | 0.83 | 0.8 | 0,01 | 0.73 | 0.72 | 0.68 | 0,01 | 0.6 | 0.64 | 0.69 | 1,21 | 35.2 | 16.4 | 8.6 | 0.90 |
| 13 | CHRM3 | 0.91 | 0.91 | 0.9 | 0,00 | 0.75 | 0.75 | 0.72 | 0,00 | 0.75 | 0.75 | 0.78 | 1,87 | 28.7 | 11.7 | 8.3 | 0.92 |
| 14 | CNR1 | 0.83 | 0.83 | 0.81 | 0,00 | 0.59 | 0.59 | 0.53 | 0,00 | 0.79 | 0.78 | 0.84 | 1,41 | 41.6 | 14.1 | 7.5 | 0.92 |
| 15 | CNR2 | 0.83 | 0.83 | 0.8 | 0,00 | 0.63 | 0.64 | 0.62 | 0,00 | 0.76 | 0.74 | 0.81 | 1,37 | 37.4 | 15.1 | 8.8 | 0.96 |
| 16 | CYP19A1 | 0.82 | 0.78 | 0.74 | 0,00 | 0.58 | 0.54 | 0.57 | 0,00 | 0.82 | 0.89 | 0.9 | 1,50 | 19.1 | 18.8 | 9.4 | 0.85 |
| 17 | DRD1 | 0.77 | 0.73 | 0.72 | 0,01 | 0.6 | 0.56 | 0.56 | 0,01 | 0.64 | 0.68 | 0.67 | 1,05 | 12.2 | 6.6 | 6.6 | 0.75 |
| 18 | DRD2 | 0.83 | 0.82 | 0.77 | 0,00 | 0.57 | 0.57 | 0.51 | 0,00 | 0.66 | 0.67 | 0.79 | 1,19 | 28.0 | 10.0 | 6.3 | 0.90 |
| 19 | EDNRA | 0.89 | 0.89 | 0.78 | 0,00 | 0.73 | 0.74 | 0.69 | -0,01 | 0.81 | 0.83 | 0.99 | 1,88 | 30.8 | 15.2 | 9.4 | 0.96 |
| 20 | ESR1 | 0.89 | 0.88 | 0.83 | 0,00 | 0.72 | 0.7 | 0.65 | 0,00 | 0.74 | 0.78 | 0.87 | 1,67 | 23.0 | 12.3 | 7.3 | 0.95 |
| 21 | HRH1 | 0.85 | 0.86 | 0.85 | 0,00 | 0.73 | 0.7 | 0.75 | 0,00 | 0.68 | 0.66 | 0.62 | 1,39 | 34.7 | 10.0 | 5.7 | 0.92 |
| 22 | HRH2 | 0.78 | 0.79 | 0.66 | 0,00 | 0.79 | 0.73 | 0.6 | 0,00 | 0.65 | 0.66 | 0.73 | 1,14 | 23.6 | 10.5 | 9.9 | 0.95 |
| 23 | HTR1A | 0.82 | 0.84 | 0.78 | 0,00 | 0.66 | 0.61 | 0.58 | 0,00 | 0.71 | 0.73 | 0.76 | 1,38 | 25.0 | 12.2 | 7.2 | 0.94 |
| 24 | HTR1B | 0.8 | 0.82 | 0.8 | 0,01 | 0.78 | 0.69 | 0.65 | 0,00 | 0.76 | 0.71 | 0.78 | 1,32 | 0.0 | 4.1 | 3.9 | 0.82 |
| 25 | HTR2A | 0.89 | 0.87 | 0.84 | 0,00 | 0.74 | 0.74 | 0.64 | -0,01 | 0.72 | 0.77 | 0.86 | 1,60 | 49.7 | 16.6 | 8.3 | 0.94 |
| 26 | HTR2B | 0.87 | 0.86 | 0.82 | -0,01 | 0.76 | 0.7 | 0.64 | 0,00 | 0.82 | 0.85 | 0.92 | 1,73 | 16.5 | 6.6 | 4.2 | 0.90 |
| 27 | HTR3A | 0.85 | 0.79 | 0.74 | 0,00 | 0.77 | 0.74 | 0.7 | 0,00 | 0.8 | 0.96 | 1.1 | 1,73 | 57.0 | 19.0 | 10.1 | 0.90 |
| 28 | KCNA5 | 0.79 | 0.8 | 0.78 | 0,00 | 0.66 | 0.65 | 0.56 | 0,00 | 0.47 | 0.45 | 0.5 | 0,80 | 29.3 | 12.2 | 9.0 | 0.92 |
| 29 | KCNH2 | 0.74 | 0.71 | 0.63 | 0,00 | 0.44 | 0.41 | 0.34 | -0,01 | 0.61 | 0.63 | 0.69 | 0,92 | 38.8 | 15.6 | 8.3 | 0.90 |
| 30 | LCK | 0.89 | 0.87 | 0.85 | -0,01 | 0.78 | 0.79 | 0.78 | 0,00 | 0.58 | 0.64 | 0.68 | 1,38 | 30.4 | 18.3 | 8.9 | 0.91 |
| 31 | MAOA | 0.78 | 0.73 | 0.66 | 0,00 | 0.55 | 0.47 | 0.43 | 0,00 | 0.66 | 0.73 | 0.78 | 1,12 | 24.9 | 16.2 | 9.3 | 0.88 |
| 32 | NR3C1 | 0.89 | 0.89 | 0.85 | 0,00 | 0.77 | 0.74 | 0.7 | -0,01 | 0.67 | 0.68 | 0.75 | 1,52 | 28.8 | 15.2 | 8.4 | 0.94 |
| 33 | OPRD1 | 0.91 | 0.9 | 0.86 | 0,00 | 0.81 | 0.78 | 0.79 | 0,00 | 0.74 | 0.77 | 0.87 | 1,89 | 9.4 | 3.7 | 3.1 | 0.90 |
| 34 | OPRK1 | 0.86 | 0.87 | 0.85 | 0,00 | 0.76 | 0.78 | 0.8 | 0,00 | 0.77 | 0.76 | 0.79 | 1,58 | 33.1 | 13.4 | 8.9 | 0.95 |
| 35 | OPRM1 | 0.89 | 0.89 | 0.89 | 0,00 | 0.75 | 0.73 | 0.7 | 0,01 | 0.74 | 0.75 | 0.81 | 1,70 | 25.0 | 15.2 | 9.1 | 0.95 |
| 36 | PDE3A | 0.91 | 0.89 | 0.85 | 0,02 | 0.74 | 0.81 | 0.76 | 0,00 | 0.53 | 0.57 | 0.59 | 1,33 | 48.3 | 19.3 | 10.2 | 0.99 |
| 37 | PDE4D | 0.95 | 0.94 | 0.91 | 0,00 | 0.88 | 0.81 | 0.77 | 0,02 | 0.58 | 0.64 | 0.73 | 1,18 | 19.2 | 20.0 | 10.0 | 0.98 |
| 38 | PPARA | 0.83 | 0.81 | 0.78 | 0,01 | 0.6 | 0.59 | 0.55 | 0,00 | 0.68 | 0.72 | 0.78 | 1,27 | 38.9 | 14.6 | 7.7 | 0.95 |
| 39 | PPARD | 0.85 | 0.85 | 0.81 | 0,01 | 0.76 | 0.73 | 0.66 | 0,00 | 0.67 | 0.67 | 0.7 | 1,34 | 22.7 | 17.7 | 9.4 | 0.96 |
| 40 | PPARG | 0.83 | 0.82 | 0.78 | 0,00 | 0.63 | 0.59 | 0.57 | 0,01 | 0.65 | 0.68 | 0.69 | 1,21 | 33.0 | 13.4 | 7.6 | 0.90 |
| 41 | PTGS1 | 0.66 | 0.63 | 0.6 | 0,00 | 0.39 | 0.39 | 0.38 | 0,00 | 0.76 | 0.78 | 0.79 | 1,04 | 25.9 | 8.4 | 5.0 | 0.77 |
| 42 | PTGS2 | 0.73 | 0.72 | 0.66 | 0,00 | 0.46 | 0.49 | 0.43 | 0,00 | 0.81 | 0.8 | 0.89 | 1,19 | 19.6 | 10.0 | 6.6 | 0.88 |
| 43 | SCN5A | 0.88 | 0.88 | 0.9 | 0,00 | 0.87 | 0.81 | 0.78 | 0,01 | 0.58 | 0.6 | 0.58 | 1,30 | 8.6 | 6.7 | 7.5 | 0.92 |
| 44 | SLC6A2 | 0.81 | 0.8 | 0.76 | 0,00 | 0.69 | 0.68 | 0.71 | 0,00 | 0.7 | 0.72 | 0.79 | 1,25 | 37.0 | 15.9 | 9.0 | 0.84 |
| 45 | SLC6A3 | 0.82 | 0.83 | 0.78 | 0,00 | 0.68 | 0.68 | 0.59 | 0,01 | 0.62 | 0.63 | 0.7 | 1,18 | 24.4 | 10.0 | 7.5 | 0.92 |
| 46 | SLC6A4 | 0.86 | 0.84 | 0.82 | 0,00 | 0.73 | 0.71 | 0.66 | -0,01 | 0.68 | 0.71 | 0.77 | 1,37 | 42.9 | 13.3 | 8.1 | 0.94 |

*Ext. = external validation, CV = 5-fold cross validation, Boot. = bootstrapping, Y. = y-scrambling, EF = enrichment factor at 1, 5, and 10 % cutoff values, AUC = area under the curve referred to hit rate.*


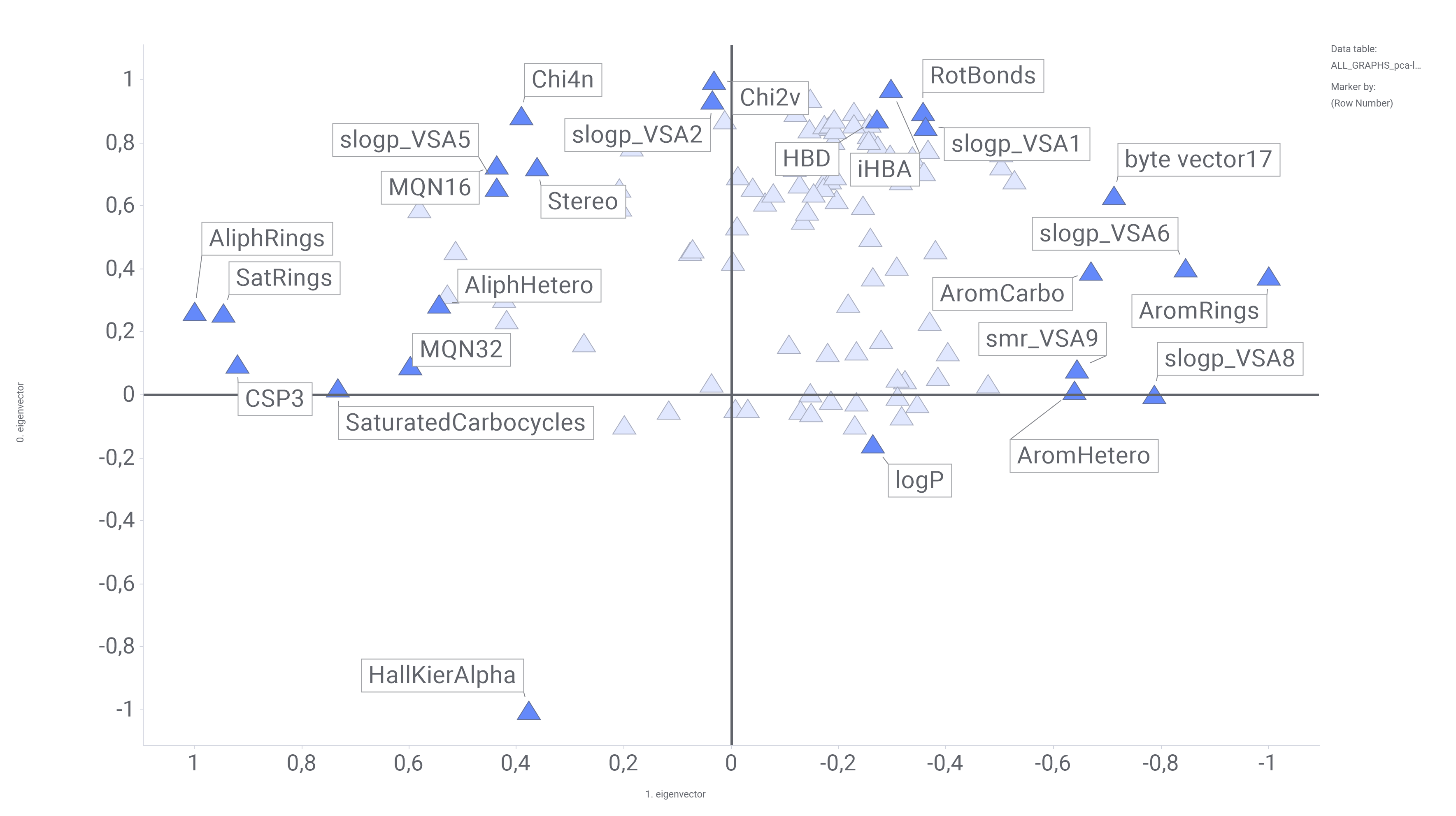


**Figure S1.** PCA loadings plot.

**Figure S2.** PCA explained variance plot.


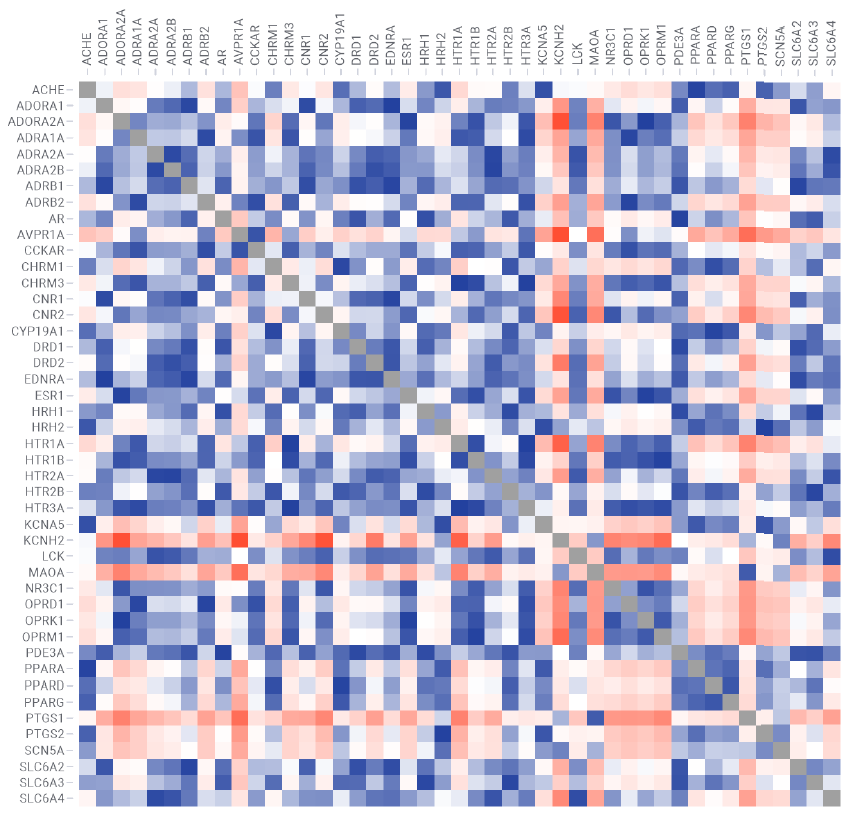


**Figure S3.** Extended version of Figure 4-a. Pairwise comparison of the 46 datasets pACTIVTY distributions computed by Kolmogorov-Smirnov statistical test. The color scale has been normalized between 0 and 1: higher values (red) indicate that the given datasets pair show significantly different distributions, as opposite to values closer to 0 (blue).


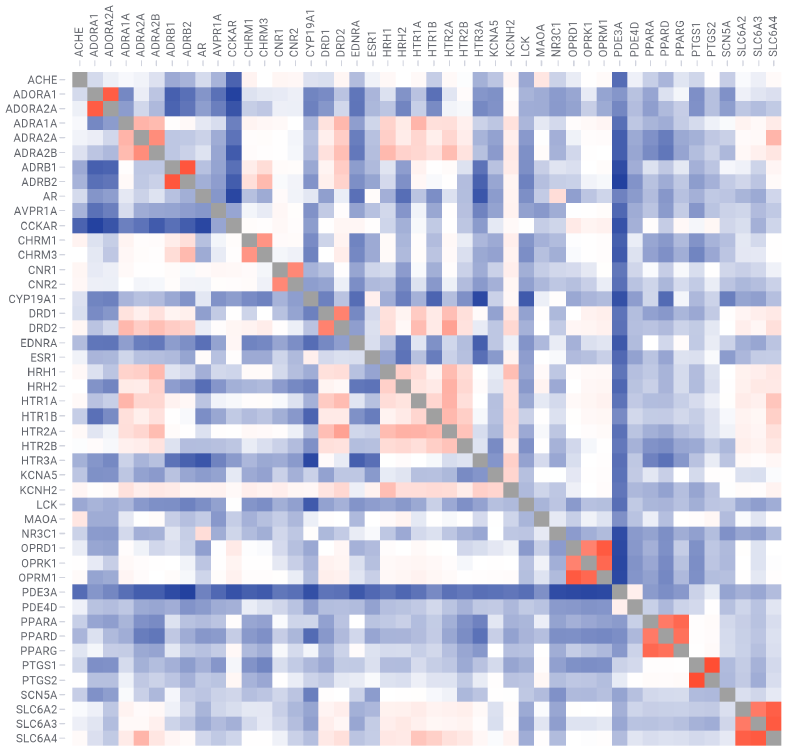


**Figure S4.** Extended version of Figure 4-b. Pairwise average Tanimoto similarity among the compounds of the given datasets pair. The color scale has been normalized between 0 and 1: higher values (red) indicate that the given datasets pair show higher chemical similarity, as opposite to values closer to 0 (blue).

**Table S3.** Dataset size, IC50/Ki measurements correlation* and performance comparison between Combined, IC50-only and Ki-only models.

| Target | Dataset size | | | IC50/Ki correlation* | | Performance | | |
| --- | --- | --- | --- | --- | --- | --- | --- | --- |
|  | ***Combined*** | ***Ki only*** | ***IC50 only*** | ***# data*** | ***R*** | ***R2_combined_*** | ***R2_Ki-only_*** | ***R2_IC50-only_*** |
| ACHE | 4759 | 436 | 3610 | 210 | 0.86 | 0.64 | 0.80 | 0.77 |
| ADORA1 | 9009 | 7036 | 546 | 219 | 0.57 | 0.56 | 0.68 | 0.51 |
| ADORA2A | 15021 | 10392 | 2048 | 185 | 0.55 | 0.67 | 0.77 | 0.80 |
| ADRA1A | 2898 | 2026 | 318 | 104 | 0.90 | 0.69 | 0.64 | 0.71 |
| ADRA2A | 3513 | 1305 | 1384 | 124 | 0.94 | 0.61 | 0.59 | 0.71 |
| ADRA2B | 2168 | 990 | 384 | 86 | 0.96 | 0.61 | 0.60 | 0.86 |
| ADRB1 | 2240 | 695 | 744 | 52 | 0.96 | 0.69 | 0.74 | 0.76 |
| ADRB2 | 4025 | 1066 | 852 | 76 | 0.83 | 0.74 | 0.60 | 0.76 |
| AR | 6585 | 1489 | 4308 | 719 | 0.03 | 0.69 | 0.68 | 0.70 |
| AVPR1A | 5878 | 2427 | 2499 | 149 | 0.73 | 0.73 | 0.62 | 0.67 |
| CCKAR | 941 | 225 | 528 | 37 | 0.95 | 0.8 | 0.65 | 0.83 |
| CHRM1 | 7576 | 2500 | 1025 | 83 | 0.86 | 0.73 | 0.57 | 0.78 |
| CHRM3 | 6886 | 3128 | 1858 | 176 | 0.34 | 0.75 | 0.81 | 0.90 |
| CNR1 | 18896 | 9031 | 5456 | 566 | 0.75 | 0.59 | 0.76 | 0.72 |
| CNR2 | 18442 | 8794 | 3205 | 441 | 0.86 | 0.63 | 0.77 | 0.76 |
| CYP19A1 | 3728 | 760 | 2514 | 372 | 0.66 | 0.58 | 0.63 | 0.60 |
| DRD1 | 2293 | 1776 | 240 | 70 | 0.73 | 0.6 | 0.67 | 0.55 |
| DRD2 | 13497 | 9506 | 2082 | 390 | 0.77 | 0.57 | 0.70 | 0.71 |
| EDNRA | 4198 | 282 | 3146 | 62 | 0.55 | 0.73 |  | 0.83 |
| ESR1 | 6659 | 700 | 4425 | 131 | 0.78 | 0.72 | 0.68 | 0.80 |
| HRH1 | 3347 | 1956 | 565 | 79 | 0.74 | 0.73 | 0.70 | 0.66 |
| HRH2 | 819 | 416 | 95 | 41 | 1.00 | 0.79 | 0.71 |  |
| HTR1A | 9021 | 6044 | 1505 | 604 | 0.53 | 0.66 | 0.72 | 0.68 |
| HTR1B | 1540 | 1164 | 241 | 23 | 0.91 | 0.78 | 0.68 | 0.47 |
| HTR2A | 11716 | 5192 | 3523 | 323 | 0.46 | 0.74 | 0.73 | 0.80 |
| HTR2B | 3283 | 1655 | 444 | 172 | 0.84 | 0.76 | 0.48 | 0.79 |
| HTR3A | 853 | 430 | 348 | 20 | 0.88 | 0.77 | 0.71 | 0.76 |
| KCNA5 | 2135 | 15 | 1649 | - | - | 0.66 |  | 0.58 |
| KCNH2 | 11325 | 1341 | 8085 | 213 | 0.63 | 0.44 | 0.64 | 0.57 |
| LCK | 3084 | 183 | 2211 | 53 | 0.84 | 0.78 |  | 0.76 |
| MAOA | 3897 | 590 | 2768 | 132 | 0.68 | 0.55 | 0.55 | 0.49 |
| NR3C1 | 10030 | 3137 | 4516 | 200 | 0.79 | 0.77 | 0.73 | 0.79 |
| OPRD1 | 7993 | 5674 | 1828 | 404 | 0.81 | 0.81 | 0.80 | 0.85 |
| OPRK1 | 9073 | 5421 | 1747 | 271 | 0.38 | 0.76 | 0.74 | 0.81 |
| OPRM1 | 13379 | 8276 | 2036 | 582 | 0.49 | 0.75 | 0.79 | 0.74 |
| PDE3A | 976 | 65 | 774 | 2 | - | 0.74 | 0.71 | 0.81 |
| PDE4D | 2710 | 69 | 2107 | 8 | - | 0.88 | 0.83 | 0.86 |
| PPARA | 6967 | 260 | 2340 | 16 | - | 0.6 | 0.46 | 0.58 |
| PPARD | 4992 | 166 | 1838 | 15 | - | 0.76 | 0.49 | 0.65 |
| PPARG | 7567 | 664 | 2244 | 56 | 0.64 | 0.63 | 0.54 | 0.73 |
| PTGS1 | 2995 | 48 | 2984 | 5 | - | 0.39 | 0.36 | 0.46 |
| PTGS2 | 6384 | 205 | 4835 | 6 | - | 0.46 | 0.42 | 0.57 |
| SCN5A | 2789 | 4 | 2216 | - | - | 0.87 |  | 0.72 |
| SLC6A2 | 7398 | 3668 | 2912 | 886 | 0.83 | 0.69 | 0.66 | 0.61 |
| SLC6A3 | 4658 | 2455 | 1983 | 502 | 0.79 | 0.68 | 0.63 | 0.64 |
| SLC6A4 | 11059 | 5157 | 5132 | 1074 | 0.73 | 0.73 | 0.77 | 0.70 |
|  |  |  |  |  |  |  |  |  |
| average |  |  |  |  | **0.73** | **0.69** | **0.66** | **0.71** |
| stdev |  |  |  |  | **0.20** | **0.10** | **0.11** | **0.11** |

**Pearson correlation between IC50 and Ki measurements when available for the same compound of a given dataset.*


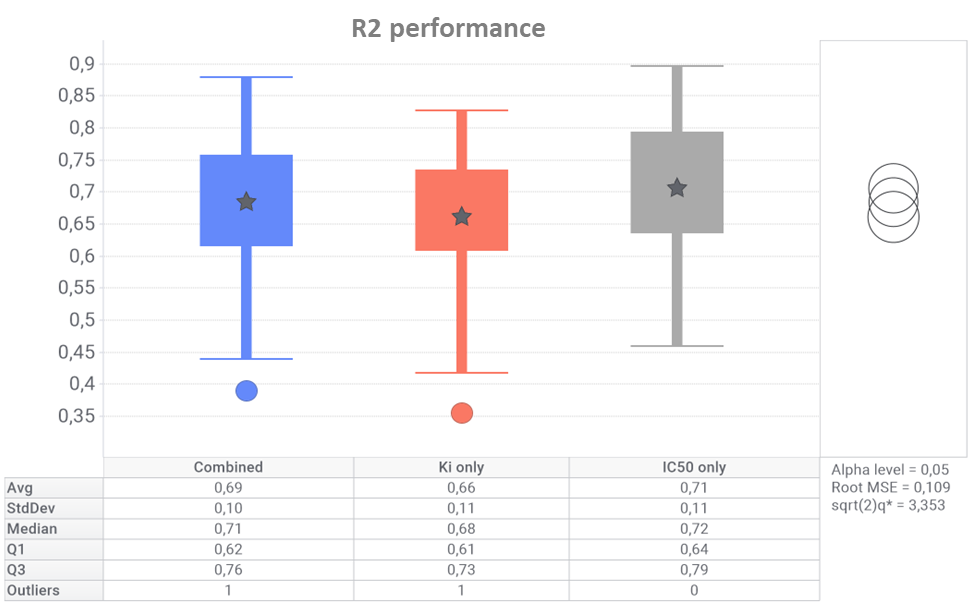


**Figure S5.** Plot of R2 performances of Table S3.


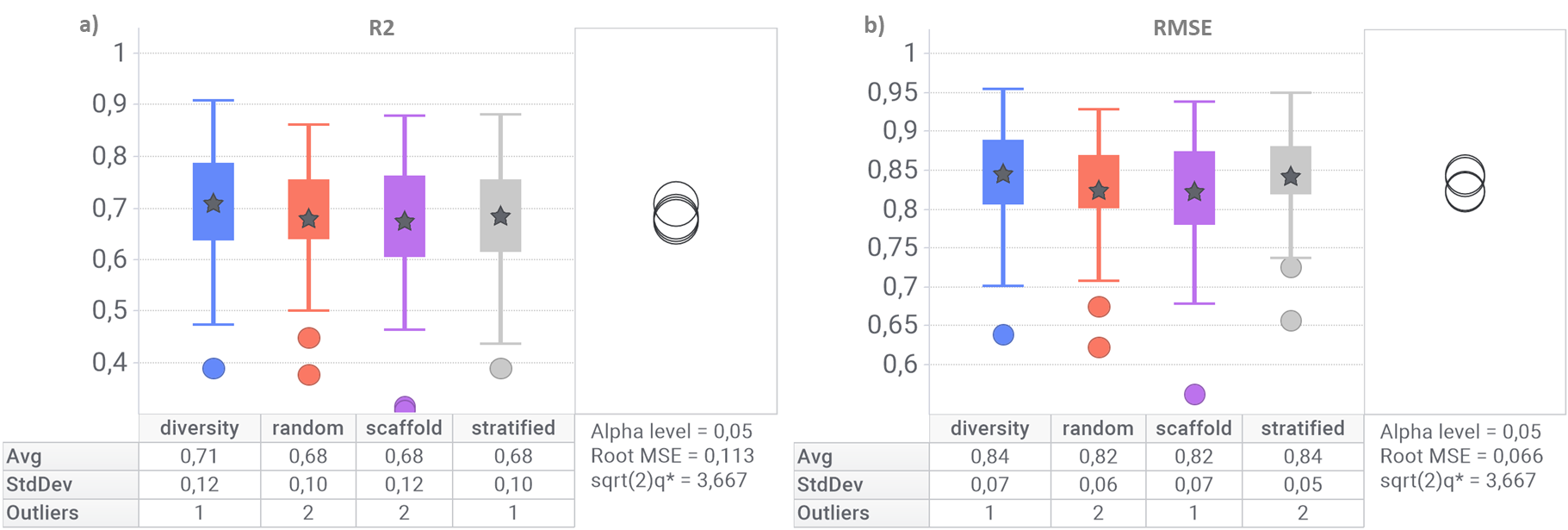


**Figure S6.** External validation performance in terms of R2 (a) and RMSE (b) according to different train/test splitting strategies. Stratified bar (grey) is referred to R2_ext_ in table S2.

**Table S4.** ProfhEX benchmarking against already published models.

| Target | ProfhEX | | | | Other works | | | | |  |
| --- | --- | --- | --- | --- | --- | --- | --- | --- | --- | --- |
|  | **RMSE** | **AUC** | **BA*** | **MCC*** | **RMSE [1]** | **AUC [2]** | **AUC [3]** | **BA [4]** | **MCC [4]** | **# molecules [4]** |
| ACHE | 0.82 | 0.97 | 0.96 | 0.91 | 0.82 | 0.91 | 0.93 | 0.88 | 0.77 | 1017 |
| ADORA1 | 0.64 | 0.95 |  |  |  | 0.95 | 0.78 | 0.82 | 0.64 | - |
| ADORA2A | 0.65 | 0.91 | 1.00 | 1.00 |  | 0.95 | 0.79 | 0.85 | 0.70 | 849 |
| ADRA1A | 0.77 | 0.94 |  |  | 0.82 | 0.88 |  | 0.78 | 0.57 | - |
| ADRA2A | 0.64 | 0.92 | 0.99 | 0.67 |  | 0.86 | 0.92 | 0.86 | 0.76 | 481 |
| ADRA2B | 0.57 | 0.95 | 1.00 | 1.00 |  | 0.90 | 0.91 | 0.76 | 0.66 | 475 |
| ADRB1 | 0.72 | 0.90 | 1.00 | 0.61 |  | 0.96 | 0.98 | 0.85 | 0.73 | 585 |
| ADRB2 | 0.71 | 0.92 | 0.99 | 0.67 |  |  | 0.66 | 0.93 | 0.85 | 570 |
| AR | 0.60 | 0.92 |  |  | 0.68 | 0.95 |  | 0.84 | 0.69 | - |
| AVPR1A | 0.62 | 0.96 | 0.99 | 0.43 |  | 0.96 |  | 0.91 | 0.82 | 538 |
| CCKAR | 0.71 | 0.91 |  |  |  | 0.99 |  | 0.89 | 0.79 | - |
| CHRM1 | 0.60 | 0.90 | 0.96 | 0.86 |  | 0.83 | 0.94 | 0.81 | 0.68 | 837 |
| CHRM3 | 0.75 | 0.92 | 0.95 | 0.88 |  | 0.91 | 0.95 | 0.90 | 0.80 | 907 |
| CNR1 | 0.79 | 0.92 | 1.00 | 0.71 | 0.74 | 0.88 |  | 0.87 | 0.75 | 811 |
| CNR2 | 0.76 | 0.96 |  |  |  | 0.90 | 0.52 | 0.83 | 0.65 | - |
| CYP19A1 | 0.82 | 0.85 |  |  |  | 0.93 |  | 0.82 | 0.63 | - |
| DRD1 | 0.64 | 0.75 | 0.50 | 0.00 |  | 0.84 | 0.78 | 0.86 | 0.74 | 573 |
| DRD2 | 0.66 | 0.90 | 0.90 | 0.54 | 0.63 | 0.88 | 0.94 | 0.84 | 0.67 | 789 |
| EDNRA | 0.81 | 0.96 |  |  |  | 0.99 |  | 0.90 | 0.85 | - |
| ESR1 | 0.74 | 0.95 | 0.91 | 0.56 | 0.68 | 0.89 | 0.61 | 0.87 | 0.72 | 552 |
| HRH1 | 0.68 | 0.92 | 1.00 | 0.91 |  | 0.92 | 0.96 | 0.87 | 0.76 | 517 |
| HRH2 | 0.65 | 0.95 | 0.98 | 0.35 |  | 0.91 | 0.93 | 0.79 | 0.70 | 470 |
| HTR1A | 0.71 | 0.94 |  |  |  | 0.90 |  | 0.83 | 0.66 | - |
| HTR1B | 0.76 | 0.82 |  |  |  | 0.92 |  | 0.88 | 0.76 | - |
| HTR2A | 0.72 | 0.94 | 1.00 | 0.70 |  | 0.88 | 0.94 | 0.85 | 0.70 | 552 |
| HTR2B | 0.82 | 0.90 | 1.00 | 1.00 |  |  | 0.92 | 0.77 | 0.57 | 504 |
| HTR3A | 0.80 | 0.90 |  |  |  | 0.90 |  | 0.91 | 0.86 | - |
| KCNA5 | 0.47 | 0.92 |  |  |  | 0.94 |  | 0.61 | 0.33 | - |
| KCNH2 | 0.61 | 0.90 | 0.92 | 0.44 | 0.62 | 0.81 | 0.91 | 0.82 | 0.64 | 1430 |
| LCK | 0.58 | 0.91 | 0.67 | 0.58 | 0.77 | 0.88 | 0.94 | 0.86 | 0.77 | 614 |
| MAOA | 0.66 | 0.88 | 0.93 | 0.80 | 0.68 | 0.94 | 0.90 | 0.82 | 0.69 | 648 |
| NR3C1 | 0.67 | 0.94 | 0.96 | 0.75 | 0.58 | 0.95 | 0.99 | 0.88 | 0.75 | 537 |
| OPRD1 | 0.74 | 0.90 | 1.00 | 1.00 |  | 0.96 | 0.43 | 0.90 | 0.80 | 559 |
| OPRK1 | 0.77 | 0.95 | 0.90 | 0.63 |  | 0.86 | 0.59 | 0.87 | 0.75 | 632 |
| OPRM1 | 0.74 | 0.95 | 1.00 | 0.71 | 0.76 | 0.89 | 0.64 | 0.88 | 0.77 | 572 |
| PDE3A | 0.53 | 0.99 |  |  |  | 0.96 |  | 0.88 | 0.79 | - |
| PDE4D | 0.58 | 0.98 |  |  |  | 0.94 |  | 0.88 | 0.76 | - |
| PPARA | 0.68 | 0.95 |  |  |  | 0.95 |  | 0.80 | 0.62 | - |
| PPARD | 0.67 | 0.96 |  |  |  | 0.96 |  | 0.84 | 0.68 | - |
| PPARG | 0.65 | 0.90 |  |  |  | 0.93 |  | 0.85 | 0.68 | - |
| PTGS1 | 0.76 | 0.77 |  |  | 0.73 | 0.93 | 0.67 | 0.65 | 0.40 | 532 |
| PTGS2 | 0.81 | 0.88 |  |  | 0.78 | 0.94 | 0.90 | 0.81 | 0.62 | 719 |
| SCN5A | 0.58 | 0.92 |  |  |  | 0.96 |  | 0.79 | 0.58 | - |
| SLC6A2 | 0.70 | 0.84 | 0.72 | 0.16 | 0.75 | 0.91 | 0.94 | 0.89 | 0.79 | 502 |
| SLC6A3 | 0.62 | 0.92 | 0.85 | 0.30 |  | 0.92 | 0.91 | 0.88 | 0.77 | 525 |
| SLC6A4 | 0.68 | 0.94 | 0.89 | 0.27 |  | 0.89 | 0.95 | 0.90 | 0.80 | 534 |

*Balanced Accuracy (BA) and Matthew’s correlation coefficient (MCC) have been recalculated on the dataset used by the authors [4], after removing compounds in ProfhEX's training set. References: [1]: 10.1186/s13321-020-00444-5; [2]: doi.org/10.1007/s10822-016-9915-2; [3]: doi.org/10.1039/C8SC00148K; [4]: doi.org/10.1186/s13321-018-0325-4

**Figure S7.** Comparison of ProfhEX performances (in terms of cross-validated RMSE) against the models published in the work Cortes-Ciriano [1].

**Figure S8.** Comparison of ProfhEX performances (in terms of cross-validated ROC AUC) against the models published in the works of Yao et al. [2] and Mayr et al. [3].

**Figure S9.** Comparison of ProfhEX performances (in terms of balanced accuracy) against the blind dataset selected from Bosc et al. [4].

**Figure S10.** Enrichment factor performance on the CDDI database. Plots a) and b) depict the enrichment factor and AUC based on liability group scores; whereas c) and d) depict the enrichment factor and AUC for the individual targets. EF has been computed at 1, 5, 10 % cutoffs.


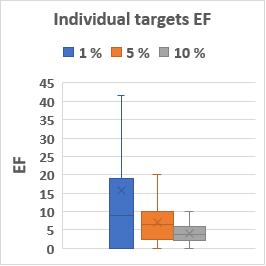


**b)**

**a)**


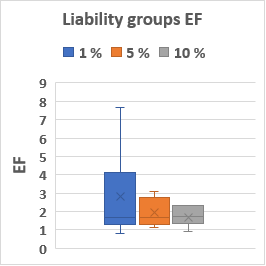


**a)**

**d)**

**Table S5.** List of 536 pre-registered, registered and withdrawn drugs taken from Cortellis Drug Discovery Intelligence database.

| CDDI_ID | Name | CDDI_ID | Name |
| --- | --- | --- | --- |
| 195612 | NA | 150595 | dalcotidine (Prop INN) |
| 276280 | methanesulfonyl fluoride | 115002 | zaltidine (Rec INN; USAN; BAN) |
| 635515 | benzgalantamine (Prop INN) memogain | 90874 | oxmetidine hydrochloride (Rec INNM; USAN; BANM) |
| 664098 | stephaglabrin sulfate stepharin sulfate | 90815 | NA |
| 462368 | 7-methoxycoumarin herniarin | 145925 | lafutidine (Rec INN) |
| 462021 | 7-acetyloxycoumarin | 90392 | nizatidine (Rec INN; USAN; BAN) |
| 325935 | NA | 161243 | pibutidine hydrochloride (Prop INNM) |
| 836726 | NA | 414232 | NA |
| 808964 | NA | 279125 | NA |
| 321347 | mimopezil (Prop INN) | 196311 | ebalzotan (Rec INN) |
| 261618 | NA | 191802 | NA |
| 215914 | anseculin hydrochloride ensaculin hydrochloride (Prop INN) | 169162 | NA |
| 160089 | NA | 142684 | binospirone mesylate (Rec INNM; USAN) |
| 153728 | suronacrine maleate (Rec INNM; USAN) | 133832 | NA |
| 163528 | metrifonate (Rec INN; USAN) metriphonate (BAN) trichlorfon | 741482 | ulotaront hydrochloride (Rec INNM) |
| 847980 | NA | 176577 | osemozotan hydrochloride (Rec INNM) |
| 705551 | neladenoson bialanate hydrochloride (Rec INN) neladenoson dalanate hydrochloride | 213595 | NA |
| 284944 | NA | 184568 | NA |
| 280140 | selodenoson (Rec INN; USAN) | 180807 | alnespirone (Rec INN) |
| 90861 | doxofylline (Rec INN; USAN) | 164608 | lesopitron dihydrochloride (Rec INNM) |
| 191356 | NA | 159809 | adatanserin hydrochloride (Rec INNM; USAN) |
| 372803 | etofylline | 136443 | zalospirone hydrochloride (Rec INNM; USAN) |
| 859723 | NA | 713091 | NA |
| 1159625 | NA | 330375 | NA |
| 1019580 | inupadenant (Rec INN; USAN) | 277810 | NA |
| 922527 | NA | 115049 | anpirtoline hydrochloride (Rec INNM) |
| 669862 | NA | 109041 | isamoltan hydrochloride (Rec INNM) |
| 959878 | etrumadenant (Rec INN; USAN) | 223823 | eletriptan (Prop INN; BAN) |
| 446515 | spongosine | 208489 | almotriptan (Prop INN; USAN) |
| 412121 | artesunate/mefloquine | 292815 | NA |
| 464910 | NA | 266815 | elzasonan hydrochloride (Rec INNM; USAN) |
| 345952 | taprizosin mesilate (Rec INNM) | 270171 | NA |
| 403404 | rezatomidine (Prop INN; USAN) | 171288 | NA |
| 90654 | centhaquin citrate (Rec INNM) centhaquine citrate (Rec INNM; USAN) | 1165828 | isoprocyn glutarate |
| 146217 | NA | 850911 | NA |
| 163683 | NA | 343987 | roluperidone hydrochloride (Rec INN; USAN) |
| 174671 | arbutamine hydrochloride (Rec INNM; USAN; BANM) | 178051 | nexopamil hydrochloride (Rec INNM) |
| 90262 | denopamine (Rec INN) | 165477 | eplivanserin fumarate (Prop INNM; USAN) |
| 115226 | bopindolol (Rec INN) | 165476 | eplivanserin mesilate (Prop INNM) |
| 273733 | NA | 164331 | serazapine hydrochloride (Rec INNM; USAN) |
| 91482 | rimoterol hydrobromide (Rec INNM; USAN; BANM; JAN) | 151484 | irindalone tartrate (Rec INNM) |
| 974880 | NA | 142901 | NA |
| 295058 | bedoradrine sulfate (Rec INNM; USAN) | 115025 | irindalone (Rec INN) |
| 188525 | deriglidole (Rec INN) | 109500 | NA |
| 177777 | picumeterol fumarate (Rec INNM; USAN; BANM) | 103705 | seganserin (Rec INN; BAN) |
| 90366 | midaglizole hydrochloride (Rec INNM) | 90537 | pelanserin hydrochloride (Rec INNM; USAN) |
| 90495 | dopexamine (Rec INN; USAN; BAN) | 163758 | carpipramine |
| 125569 | bitolterol mesylate (Rec INNM; USAN; BANM) | 283381 | NA |
| 90622 | mabuterol hydrochloride (Rec INNM; USAN) | 163763 | eplivanserin (Prop INN; USAN) |
| 91485 | pirbuterol hydrochloride (Rec INNM; USAN; BANM) | 339801 | NA |
| 91080 | tulobuterol hydrochloride (Rec INNM; BANM) | 469048 | metadoxine pyridoxine pyrolate |
| 91483 | reproterol hydrochloride (Rec INNM; USAN; BANM) | 440286 | ethenzamide |
| 835192 | hexoprenaline sulfate | 251853 | pinoline |
| 439329 | abediterol (Prop INN; USAN) | 468503 | NA |
| 250620 | NA | 170837 | besipirdine hydrochloride (Rec INNM; USAN) |
| 207891 | (R)-tulobuterol meluadrine tartrate (Prop INNM) tulobuterol-(R) | 322305 | atecegatran (Prop INN) |
| 337014 | trantinterol hydrochloride | 148750 | besipirdine (Rec INN; USAN) |
| 308981 | salmefamol (Rec INN; BAN) | 441182 | NA |
| 254077 | NA | 314607 | 20(S)-ginsenoside Rg3 20(S)-propanaxidiol ginsenoside Rg3 |
| 250616 | NA | 763054 | narazaciclib (Prop INN) |
| 211498 | NA | 442545 | NA |
| 467215 | NA | 115032 | esuprone (Rec INN) |
| 170915 | NA | 689234 | contezolid (Rec INN; USAN) |
| 153454 | NA | 917550 | contezolid acefosamil (USAN) |
| 654572 | NA | 90470 | brofaromine hydrochloride (Rec INNM) |
| 629189 | vosilasarm (Rec INN) | 301749 | NA |
| 433856 | dimethandrolone undecanoate | 672647 | delpazolid (Rec INN) |
| 236954 | NA | 439503 | NA |
| 770541 | peruvoside | 126384 | NA |
| 126393 | osaterone acetate (Rec INNM) | 266743 | afobazole fabomotizole hydrochloride (Rec INNM) mobazol obenoxazine hydrochloride |
| 888892 | deutenzalutamide (Prop INN; USAN) | 254133 | NA |
| 843689 | rezvilutamide (Rec INN) | 194029 | NA |
| 385516 | anhydroicaritin icaritin | 162126 | NA |
| 340989 | diindolylmethane | 104031 | 20-O-beta-D-glucopyranosyl-20(S)-protopanaxadiol ginsenoside C-K ginsenoside K ginsenoside M1 panaxoside |
| 129426 | zanoterone (Rec INN; USAN) | 1140398 | NA |
| 111462 | cioteronel (Rec INN; USAN) X-andron | 954595 | NA |
| 90413 | bifluranol (Rec INN; BAN) | 887002 | zavacorilant (Rec INN) |
| 1000128 | bavdegalutamide (Rec INN; USAN) | 91049 | trilostane (Rec INN; USAN; BAN) |
| 868554 | ralaniten acetate (Prop INNM; USAN) | 657697 | mapracorat (Prop INN; USAN) |
| 640472 | NA | 287000 | prednisolone sodium metasulfobenzoate prednisolone sodium metazoate (USAN) |
| 396526 | NA | 113627 | tipredane (Rec INN; USAN; BAN) |
| 749119 | NA | 281034 | dexamethasone sodium phosphate |
| 699042 | seviteronel (Rec INN) | 394522 | NA |
| 472624 | ligandrol | 307322 | etiprednol dicloacetate (Rec INN; USAN) |
| 174825 | turosteride (Rec INN) | 254573 | zoticasone propionate (Rec INNM) |
| 116550 | inocoterone acetate (USAN) | 213088 | NA |
| 115398 | tibolone (Rec INN; USAN; BAN) | 166657 | butixocort (Rec INN) |
| 690254 | testosterone beta-cyclopentylpropionate testosterone cypionate | 107010 | butixocort propionate tixocortol buryrate propionate |
| 344083 | fluridil topilutamide (Prop INN) | 333442 | dexamethasone cipecilate (Rec INN) |
| 306576 | oxymetholone | 170014 | loteprednol etabonate (Rec INNM; USAN) |
| 788152 | proxalutamide pruxelutamide (Rec INN) | 154424 | prednisolone farnesylate |
| 730297 | darolutamide (Rec INN; USAN) | 129623 | methylprednisolone suleptanate (Rec INN; USAN) |
| 145770 | finasteride (Rec INN; USAN; BAN) | 136805 | rimexolone (Rec INN; USAN; BAN) trimexolone |
| 306300 | testosterone undecanoate (USAN) | 170262 | betamethasone butyrate propionate (Rec INNM) |
| 91422 | oxandrolone (Rec INN; USAN; BAN; JAN) | 164968 | deprodone propionate (Rec INNM) |
| 837602 | NA | 100332 | methylprednisolone aceponate (Rec INN) |
| 789731 | NA | 168221 | halobetasol propionate (USAN) ulobetasol propionate (Rec INNM) |
| 785933 | NA | 111914 | dexamethasone palmitate (Rec INNM) |
| 919862 | cortexolone 17alpha-valerate-21-propionate | 90741 | halopredone acetate (Rec INNM; USAN) |
| 839129 | ribuvaptan (Rec INN) | 115029 | hydrocortisone aceponate (Rec INN) |
| 234434 | NA | 117492 | prednicarbate (Rec INN; USAN) |
| 176915 | NA | 127125 | halometasone (Rec INN) |
| 161097 | pranazepide (Prop INN) | 125574 | alclometasone dipropionate (Rec INN; USAN; BAN) |
| 927253 | NA | 287439 | betamethasone dipropionate |
| 327775 | NA | 284151 | triamcinolone hexacetonide (Prop INN; USAN; BAN) |
| 205396 | tazomeline (Prop INN; USAN) | 553343 | fludroxycortide flurandrenolide |
| 167179 | thiopilocarpine | 91424 | prednisolone acetate (Rec INNM; USAN; BANM; JAN) |
| 149652 | itameline (Rec INN) | 70003 | methylprednisolone |
| 124025 | NA | 823391 | fluorometholone acetate |
| 704615 | NA | 450114 | fluorometholone |
| 144179 | nebracetam fumarate (Rec INNM) | 399775 | cortivazol |
| 105193 | nebracetam (Rec INN) | 395990 | difluprednate (USAN) |
| 902277 | NA | 336436 | hydrocortisone probutate |
| 175663 | NA | 333901 | prednisolone sodium phosphate (USAN; JAN) |
| 152579 | NA | 317990 | hydrocortisone acetate (Rec INNM; BANM) |
| 266487 | imidafenacin (Rec INN) | 305717 | fluocortin butyl (Prop INNM; USAN; BANM) |
| 91544 | dicyclomine hydrochloride (USAN; BANM) dicycloverine hydochloride (Rec INNM; JAN) | 305559 | diflorasone (Prop INN; BAN) |
| 938871 | abeprazan hydrochloride fexuprazan hydrochloride (Prop INNM) | 305557 | cloprednol (Prop INN; BAN) |
| 1054754 | 4-[18F]fluorodexetimide | 282612 | betamethasone 17-valerate betamethasone valerate (USAN; BAN; JAN) |
| 963602 | NA | 282593 | triamcinolone (Prop INN; USAN; BAN; JAN) |
| 167560 | penehyclidine penehyclidine hydrochloride penethequinine | 279288 | methylprednisolone sodium succinate |
| 100346 | tenilapine (Prop INN) | 274937 | desonide (Prop INN; USAN; BAN) |
| 116116 | nipenzepine nuvenzepine (Rec INN) | 91627 | clobetasone butyrate |
| 193155 | temiverine hydrochloride hydrate (Prop INNM) | 91625 | halcinonide |
| 322000 | NA | 91623 | clocortolone pivalate (USAN) |
| 195836 | revatropate (Prop INN; BAN) | 90004 | naflocort (Rec INN; BAN) |
| 906006 | NA | 90101 | azacort deflazacort (Rec INN; USAN; BAN) oxazacort |
| 337120 | afacifenacin fumarate (Prop INNM) | 91614 | fluocinolone acetonide (Rec INN; USAN; BAN; JAN) |
| 634946 | NA | 652408 | hydrocortisone 17-butyrate |
| 70520 | oleamide | 473607 | dexamethasone 21-acetate dexamethasone acetate |
| 327197 | dronabinol hemisuccinate | 322798 | betamethasone sodium phosphate (Rec INNM; USAN; BANM; JAN) |
| 314401 | voacamine voacanginine | 91622 | clobetasol propionate |
| 636826 | NA | 636203 | fosdagrocorat (Rec INN; USAN) |
| 552274 | cannabidiolic acid | 315603 | zoticasone (Rec INN) |
| 723475 | [11C]cetrozole | 160459 | NA |
| 149510 | liarozole hydrochloride (Rec INNM; USAN; BANM) | 153873 | NA |
| 141393 | liarozole (Rec INN; BAN) | 335391 | NA |
| 100903 | atamestane (Prop INN) | 294488 | NA |
| 271217 | vatalanib succinate (Rec INNM; USAN) | 926210 | NA |
| 209703 | liarozole fumarate (Rec INNM; USAN; BANM) | 399047 | NA |
| 895368 | testololactone | 940827 | amdakefalin (Rec INN) |
| 239171 | NA | 173614 | semorphone hydrochloride (Rec INNM) |
| 197055 | NA | 141247 | NA |
| 188157 | odapipam acetate (Prop INNM) | 949722 | NA |
| 785794 | NA | 163822 | fedotozine tartrate (Rec INNM) |
| 231811 | NA | 207757 | NA |
| 222577 | adrogolide hydrochloride (Prop INNM; USAN) | 163789 | apadoline (Prop INN) |
| 221059 | NA | 123888 | NA |
| 211718 | berupipam maleate (Rec INNM) | 401364 | dinalbuphine sebacate (Prop INN) dinaphine sebacoyl dinalbuphine ester |
| 193864 | NA | 895216 | NA |
| 170985 | zelandopam hydrochloride (Rec INNM) | 855575 | NA |
| 111845 | NA | 308951 | oxilorphan (Rec INN; USAN) |
| 102608 | deoxyepinephrine epinine N-methyldopamine | 812770 | asalhydromorphone hydrochloride (Rec INNM) |
| 158398 | dopexamine hydrochloride (Rec INNM; USAN; BANM) | 462733 | buprenorphine hemiadipate |
| 129206 | ibopamine (Rec INN; USAN; BAN) | 105694 | dalargin |
| 157479 | NA | 815838 | NA |
| 308481 | clopimozide (Rec INN; USAN) | 654616 | NA |
| 127684 | NA | 251677 | frakefamide (Rec INN) |
| 1075216 | NA | 452175 | naloxegol (Prop INN; USAN) PEG-naloxol pegylated naloxol |
| 785756 | [18F](N-methyl)benperidol | 185000 | remifentanil hydrochloride (Rec INNM; USAN; BANM) remifentanyl hydrochloride |
| 655731 | NA | 422210 | piritramide |
| 449372 | NA | 299650 | tilidine hydrochloride (Prop INNM; USAN; BANM) |
| 271569 | abaperidone hydrochloride (Prop INNM) | 90803 | dihydrocodeine bitartrate (Prop INNM; USAN) |
| 200919 | NA | 91093 | alfentanil hydrochloride (Rec INNM; USAN; BANM) |
| 175466 | carvotroline hydrochloride (Rec INNM; USAN) | 91009 | propiram fumarate (Rec INNM; USAN; BANM) |
| 149374 | [123I]iolopride (Prop INNM) | 1007703 | NA |
| 147145 | elopiprazole (Rec INN) | 309198 | nafenopin (Prop INN; USAN) |
| 136701 | NA | 292314 | oxeglitazar (Rec INN) |
| 104354 | NA | 714395 | icosabutate (Rec INN; USAN) |
| 930540 | NA | 265033 | NA |
| 765506 | dordaviprone dihydrochloride (Prop INNM) | 90802 | binifibrate (Rec INN) binifibrato |
| 235902 | nolomirole hydrochloride (Rec INNM) | 122952 | ronifibrate (Prop INN) ronifibrato |
| 150388 | NA | 91117 | clinofibrate (Rec INN) |
| 121397 | NA | 356113 | etofibrate (Prop INN) |
| 441006 | NA | 308979 | etofilline clofibrate (Prop INN) |
| 441003 | NA | 802540 | saroglitazar magnesium (Rec INNM) |
| 971439 | deudomperidone (Rec INN; USAN) | 454150 | NA |
| 809665 | [123I]epidepride | 417672 | cevoglitazar (Rec INN) |
| 433274 | NA | 346692 | NA |
| 348767 | NA | 340606 | fonadelpar (Rec INN; USAN) |
| 227533 | piricapiron | 1022790 | mavodelpar sodium (Prop INNM) |
| 194173 | mazapertine (Rec INN; USAN) | 963492 | NA |
| 175574 | cipazoxapine savoxepin mesylate (Rec INNM) | 447150 | NA |
| 140092 | etrabamine hydrochloride (Rec INNM) | 154417 | darglitazone (Rec INN; USAN) |
| 129622 | cipazoxapine savoxepin (Rec INN) | 284395 | bardoxolone methyl (Prop INNM; USAN) |
| 90704 | NA | 1101187 | NA |
| 723051 | aripiprazole laurate aripiprazole lauroxil (USAN) | 773315 | NA |
| 161247 | perospirone hydrochloride (Rec INNM) | 197570 | etalocib sodium (Rec INNM; USAN) |
| 90433 | isofloxythepin | 194140 | NA |
| 91475 | alizapride (Rec INN) | 211578 | adelmidrol (Prop INN) |
| 91070 | veralipride (Rec INN) | 354505 | sipoglitazar (Rec INN) |
| 91415 | pipothiazine (BAN) pipotiazine (Rec INN) | 283575 | edaglitazone (Rec INN; USAN) |
| 433920 | fluphenazine decanoate | 167937 | madecassoside |
| 91046 | clopenthixol hydrochloride zuclopenthixol hydrochloride (Rec INNM; BANM) | 147690 | NA |
| 305485 | timiperone (Prop INN; JAN) | 373614 | glycyrin |
| 165804 | iloperidone (Rec INN; USAN) | 925930 | NA |
| 388735 | paliperidone palmitate (USAN) | 275734 | benoxaprofen |
| 100826 | quinagolide hydrochloride (Rec INNM; BANM) | 305558 | parsalmide (Prop INN) |
| 394639 | thiethylperazine dimaleate | 139569 | salmisteine (Rec INN) |
| 1023263 | trazpiroben maleate (Prop INNM; USAN) | 1039542 | ibuprofenamine hydrochloride |
| 386199 | perphenazine 4-aminobutyrate mesylate | 178932 | parcetasal (Rec INN) |
| 293826 | NA | 320676 | naproxen Etemesil (USAN) |
| 757464 | N-[11C]methylbenperidol | 197388 | salnacedin (Prop INN; USAN) |
| 650034 | NA | 113665 | indomethacin butanediol ester |
| 328797 | biriperone centbutindole | 90364 | tilomisole (Rec INN; USAN) |
| 308580 | milenperone (Prop INN; USAN) | 90201 | itazigrel (Rec INN; USAN) itazogrel |
| 279345 | tezosentan disodium (Rec INNM) | 149069 | amtolmetin guacil (Rec INN) |
| 281453 | NA | 90323 | indometacin farnesil (Rec INNM; JAN) indomethacin farnesil (USAN; BANM) |
| 239030 | clazosentan (Rec INN; USAN) | 90456 | tropesin |
| 258492 | clazosentan sodium (Rec INNM; USAN) | 142648 | guaimesal (Rec INN) |
| 208304 | NA | 91040 | ibuprofen guaiacol ester (Rec INNM) methoxybutropate metoxibutropate |
| 1078735 | imlunestrant (USAN) | 115082 | fosfosal (Rec INN) |
| 982161 | giredestrant (Rec INN; USAN) | 91058 | ibuprofen piconol pineprofen |
| 1124261 | vepdegestrant (Prop INN) | 91052 | acemetacin (Rec INN; BAN) |
| 91043 | norgestimate (Rec INN; USAN; BAN) | 281469 | fenbufen |
| 300721 | 17-deacetylnorgestimate levonorgestrel 3-oxime norelgestromin (Rec INN) | 91565 | bismuth salicylate bismuth subsalicylate (USAN) |
| 1059346 | NA | 91590 | acetylsalicylic acid lysine salt lysine acetylsalicylate |
| 189569 | NA | 305489 | proglumetacin maleate |
| 144686 | NA | 340462 | NA |
| 144684 | NA | 147137 | NA |
| 131059 | clofurenadine hydrochloride | 363426 | imrecoxib |
| 91438 | iprindole (Rec INN; USAN; BAN) | 722603 | 15-hydroxyeicosatrienoic acid |
| 214980 | efletirizine (Rec INN; USAN) | 317647 | NA |
| 137356 | tazifylline hydrochloride (Rec INNM; USAN) | 191296 | darbufelone (Prop INN) |
| 129844 | tagorizine (Rec INN) | 280412 | apricoxib (Prop INN; USAN) |
| 103709 | fenclozine maleate | 224688 | NA |
| 91259 | picumast dihydrochloride (Rec INNM; BANM) | 144126 | NA |
| 634054 | NA | 295843 | polmacoxib (Prop INN; USAN) |
| 236612 | NA | 117301 | aceclofenac (Rec INN; USAN; BAN) |
| 200676 | NA | 269105 | nepafenac (Prop INN; USAN) |
| 193871 | mapinastine maleate (Rec INNM) | 334529 | NA |
| 193162 | flezelastine hydrochloride (Rec INNM) | 91523 | moracizine hydrochloride (Rec INNM; BANM) moricizine hydrochloride (USAN) |
| 162120 | pibaxizine (Rec INN) | 91091 | isocainide hydrochloride lorcainide hydrochloride (Rec INNM; USAN; BANM) socainide hydrochloride |
| 144471 | noberastine (Rec INN; USAN; BAN) | 236882 | NA |
| 103635 | rocastine fumarate (Rec INNM) | 182089 | silperisone hydrochloride (Prop INN) |
| 107839 | levocabastine hydrochloride (Rec INNM; USAN; BANM) | 955884 | ammoxetine ammuxetine |
| 108652 | levdropropizine levodropropizine (Prop INN) | 643030 | deuterated paroxetine |
| 127863 | setastine hydrochloride (Rec INNM) | 477408 | nortriptyline-GABA |
| 327067 | doxylamine succinate (Prop INNM; USAN; BANM) | 454427 | NA |
| 310003 | carbinoxamine maleate (Prop INNM; USAN; BANM) | 161109 | NA |
| 307255 | betahistine dihydrochloride (USAN) | 707693 | [18F]fluorobenguan flubrobenguane (18F) (Rec INN) flubrobenguane F18 (USAN) |
| 133511 | elbanizine (Rec INN) | 818804 | [18F]-meta-fluorobenzylguanidine |
| 194185 | NA | 90353 | clovoxamine (Rec INN) |
| 191770 | NA | 635980 | edivoxetine hydrochloride (USAN) |
| 125789 | NA | 401184 | NA |
| 117800 | donetidine (Rec INN; USAN; BAN) | 401183 | NA |
| 135503 | ebrotidine (Rec INN) | 384106 | NA |
| 159328 | osutidine (Prop INN) | 384102 | NA |
| 155963 | NA | 384099 | NA |
| 246816 | [99mTc]technetium tropantiol technetium (99mTc) tropantiol (Prop INNM; USAN) | 384098 | NA |
| 921497 | armesocarb | 384078 | NA |
| 776572 | NA | 384077 | NA |
| 285766 | delucemine hydrochloride (Prop INNM; USAN) | 384076 | NA |
| 158612 | moxifetin hydrogen maleate | 384074 | NA |
| 136023 | cericlamine hydrochloride (Rec INNM) | 384073 | NA |
| 90185 | femoxetine (Rec INN) | 821981 | NA |
| 757557 | NA | 392789 | NA |
| 457292 | NA | 264071 | NA |
| 882590 | pirepemat | 229061 | manifaxine hydrochloride (Rec INNM) |
| 148608 | (+)-dapoxetine hydrochloride (Rec INNM; USAN) dapoxetine dapoxetine hydrochloride | 214896 | neboglamine (Rec INN) nebostinel |
| 117042 | indeloxazine hydrochloride (Rec INN; USAN) | 174633 | napitane mesilate (Prop INNM; USAN) |
| 90068 | clopradone etoperidone hydrochloride (Rec INNM; USAN) triazolinone | 172645 | NA |
| 327903 | alaproclate (Rec INN; USAN) | 377425 | L-lysine-d-amphetamine dimesylate lisdexamfetamine dimesylate (USAN) lisdexamfetamine mesilate (Rec INNM; USAN) |
| 91005 | iobenguane I 131 (USAN) iobenguane[131I] (Rec INN) | 836196 | (R)-phenotropil (R)-phenylpiracetam |
| 328128 | nitroxazepine hydrochloride | 275397 | mesocarb (Prop INN) sydnocarbum |
| 821392 | netarsudil mesylate (Rec INNM; USAN) | 190995 | [123I]iometopane (Prop INN; USAN) |
| 760157 | NA | 90128 | binedaline (Rec INN) binodalina |
| 680372 | fenotropil fonturacetam (Prop INN) phenylpiracetam | 224675 | brasofensine sulfate (Prop INNM) |
